# Accessible data collection methods in clinical trials: do current best practices for the implementation of electronic patient-reported outcome measures (ePROMs) meet accessibility standards?

**DOI:** 10.1186/s41687-026-01039-8

**Published:** 2026-03-25

**Authors:** Florence D. Mowlem, Paul O’Donohoe

**Affiliations:** 1 uMotif, London, UK; 2 Medidata Solutions, London, UK

**Keywords:** Accessibility, ePRO, eCOA, Diversity, Burden, Migration

## Abstract

Apps and web browsers, whether on smartphones, tablets or laptops, have become the mainstream methods of capturing clinical outcome assessment (COA) data, and patient-reported outcome measures (PROMs) in particular, in clinical trials. There has long been concern around whether implementing traditionally paper-based questionnaires on electronic systems may impact the measurement properties of these carefully validated questionnaires, with migration best practices focusing predominately on this issue. In parallel, app and web-design best practices outside of clinical research have focused on the accessibility and usability of these electronic systems for the widest range of users. This article evaluates how existing web accessibility success criteria compare to electronic PROM (ePROM) design best practices, identifying where there is alignment or tension, where further evidence is needed to ensure both accessibility and the maintenance of the questionnaire measurement properties, and what accessibility practices can be incorporated into ePROM design best practices today.

**Supplementary Information:**

The online version contains supplementary material available at 10.1186/s41687-026-01039-8.

## Introduction

Accessibility encompasses the extent to which products and services for a specific context of use can be used by individuals with the widest range of characteristics and capabilities possible [1]. It is a key element of supporting diversity, equity, and inclusion; without accessibility, these cannot truly be achieved as certain subgroups will be excluded from easily engaging with the products and services [2]. While accessibility of app- and web-based solutions for *all* potential users has long been a driving force of design outside clinical research, this article focuses on accessibility in the context of the electronic capture of data from patient-reported outcome measures (PROMs). In the last 20 years, electronic capture of clinical outcome assessments (COAs), in particular PROMs (ePROMs), utilizing web- and app-based systems, has become the mainstream method of data collection in clinical trials due to the benefits offered compared to pen-and-paper [3].

The importance of accessibility and usability in PROM development and implementation is starting to be recognized by regulators [4, 5]. Specifically, in their most recent patient focused drug development (PFDD) guidance series, the US Food and Drug Administration (FDA) addresses the benefit of “universal design” for COAs and the importance of ensuring accessible data collection methods, with specific reference to electronic data capture and assistive technologies. This means ensuring the design and composition of a COA enable it to be accessed, understood, and used to the greatest extent possible by all people, including those with disabilities, and can facilitate broad inclusion in trials. [5].

Patients, like the general population, may be impacted by impaired functioning in a range of domains that can affect their interaction with data collection methods, including impaired cognitive function, vision impairment, impaired fine motor control, low literacy, and learning difficulties. While such challenges may have been life-long and independent of their reasons for being engaged in clinical research, these patients face the additional challenge that such issues *may* be a symptom of their condition or a side-effect of treatment, and can impact their use of technology for completing PROMs [6] within the context of attempting to better understand that condition or treatment. To date, best practices for developing and implementing ePROMs has largely ignored accessibility as understood in the context of universal design, arguably to the significant detriment of clinical research and, more importantly, patients.

In their PFDD Guidance, FDA direct the reader to review the World Wide Web Consortium (W3C) Web Accessibility Initiative (WAI) recommendations, as well as Sect.  508 of the US Government website (that requires Federal agencies to ensure that their Information and Communication systems are accessible to individuals with disabilities, in a way that is comparable to those without disabilities) to ensure a PROM will be accessible for individuals with certain impairments [5]. However, to date there has not been broad adoption, nor consideration of how this may create a tension with existing ePROM design best practices, which are also referenced in the same guidance.

The aim of this article is to review the web accessibility success criteria through the lens of ePROM design and implementation best practices to identify points of alignment and points of incongruence. We begin by providing a brief description of web accessibility criteria and history of ePROM design best practices to set the context for the comparison between the two. Considerations will be provided for how accessibility success criteria might be incorporated into future ePROM best practices and where further research is recommended to demonstrate the maintenance of the measurement properties of the ePROM, pointing towards a future of clinical research solutions which not only ensure high quality data capture, but are also accessible and supportive for the greatest array of participants, ultimately giving us greater confidence in our understanding of novel treatments.

### Accessibility guidelines

**Fig. 1:**
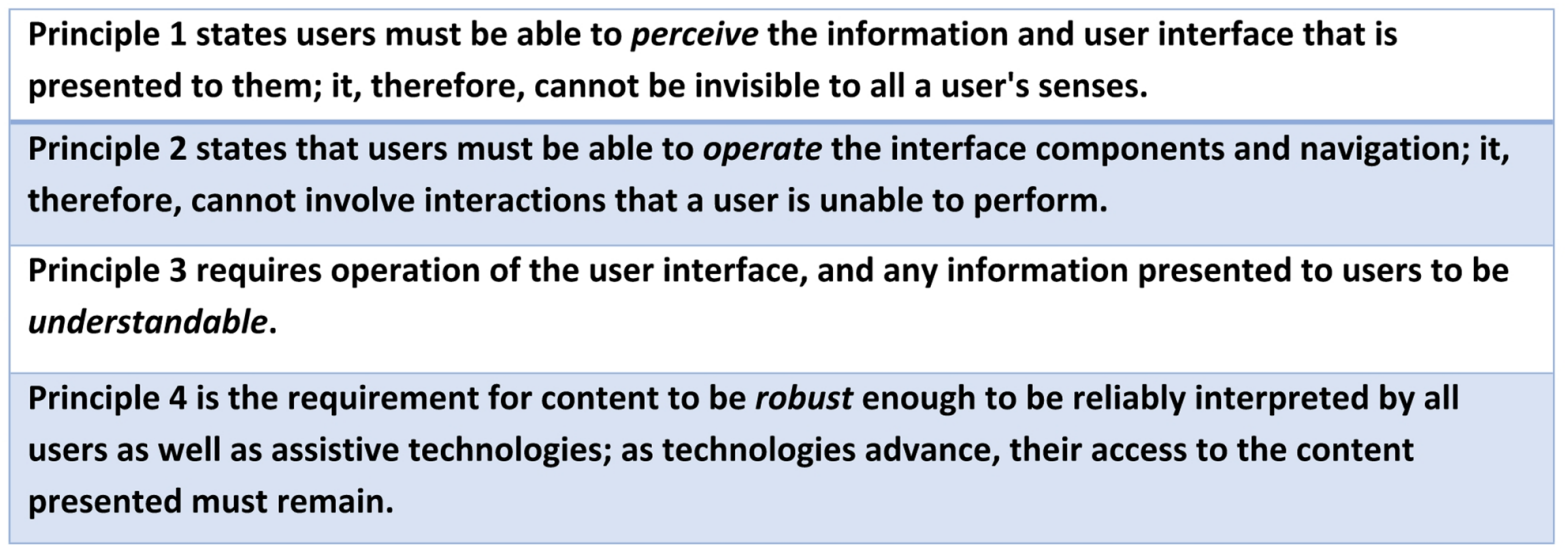
The four web accessibility guideline principles from W3C [7]

Accessibility of web solutions is usually evaluated through a conformance assessment based on the W3C Web Content Accessibility Guidelines (WCAG 2.2) [7], which list 13 guidelines organized around 4 principles (see Fig. 1.), with corresponding success criteria that are classified into 3 levels, from lowest to highest conformance (A, AA, AAA) [7]. All four principles must be true for users with disabilities to be able to readily use the web. These four principles are also outlined in EU legislation to ensure websites and mobile applications are accessible [8].

W3C acknowledge that whilst there are some slightly different considerations for accessibility on mobile devices (i.e., smartphones and tablet devices) as compared to desktop/laptop, there is no absolute divide and common features exist (e.g., both can include touchscreen control, the use of external keyboards, and responsive design), and so “Overall, **WCAG 2.0 is highly relevant to both web and non-web mobile content and applications**” [9]. Nevertheless, W3C published discussion of mobile-related issues to support accessibility, with additional best practices where needed. This is relevant to ePROMs, which are predominately deployed using app and web on mobile devices, and to a lesser degree desktop/laptop.

### ePROM best practices

Historically, best practices for the implementation of ePROMs has focused on the issue of “faithful migration” [10, 11] and ensuring electronic implementations of originally paper-based questionnaires capture comparable data. This is generally a story of concern about minor changes in visual layout potentially leading to participants responding differently to questions about how they are feeling and functioning. This concern was largely driven by the 2009 FDA Guidance on the use of PROMs in medical product labelling [12], which stated that *“When a PRO instrument is modified*,* sponsors generally should provide evidence to confirm the new instrument’s adequacy”* and explicitly referred to modification including *“changing an instrument from paper to electronic format”* (pp. 20). An ISPOR Task Force Report [13] expanded on this statement, recommending that testing is conducted to confirm the comparability between modes of administration. These recommendations had a lasting influence.

In the 15 years since these seminal publications, large amounts of research have repeatedly demonstrated the comparability of PROMs when migrating from paper to electronic format for common item and response scale types [14–19], in addition to measurement comparability between electronic formats for bring-your-own-device (BYOD) implementations [17, 18, 20]. This body of research has culminated in updated industry recommendations that further studies of measurement comparability are not needed in cases where modifications to the migrated questionnaire are minor-to-moderate, sufficient comparability evidence exists, and importantly, best practices for migration are followed [11].

The historical focus on mitigating the risk of non-comparability is understandable given these measures often support key endpoints in pivotal trials and play a role in the ultimate success (or otherwise) of bringing a new compound to market. However, this has incentivized the pursuit of a reduction in variability in layout and presentation of content, and has meant that, in general, electronic applications for capturing PROMs have remained staunchly inflexible in design and layout, and not prioritized (or considered at all) inclusive design principles.

### Shifting focus

Arguably, this historic focus has been at the expense of optimizing the user experience of interacting with the technology, and the industry has been slow to explore alternative ways of presenting PROMs on screen-based devices – presentations that are often very common in other contexts. For example, it is only recently the first investigation on the impact of scrolling on ePROM implementations was published [20]. Scrolling has long been used in non-ePROM contexts to allow for the presentation of content without having to reduce font size to an unusable degree, but has historically been advised against in ePROMs due to concerns that if users are required to scroll to see all response options it may bias their responses [21]. While Shahraz et al. [20] found scrolling did not negatively impact the measurement properties, they still conservatively recommended the avoidance of scrolling. More generally, measures developed in electronic format ab initio still tend to follow existing best practices despite their primary focus being on migration from paper format, rather than taking full advantage of their screen-based modes of data capture and the flexibility this allows in making content accessible for the widest range of users.

As an industry, we must hold ourselves accountable and challenge ourselves to consider whether the desire to strictly control how PROMs are administered and implemented on electronic modes of data capture due to a fear of negatively impacting measurement properties, has actually led to suboptimal solutions which are not as user-friendly and accessible for a broad range of individuals. As a consequence, this raises the concern of whether we are collecting data in a suboptimal manner from non-representative populations, reducing the utility of data in a trial, and, ultimately, risking the success of bringing a new compound to market.

It is worth noting that the adaptability of screen-based designs affords us the opportunity to make content *more* accessible and usable. Traditional paper-and-pen lack any of this flexibility and can be extremely difficult, if not impossible, for certain populations to use. However, moving from paper-and-pen to electronic capture of PROMS, while offering a wealth of benefits, can still create barriers for some individuals if not approached thoughtfully.

Whilst the recent updated best practices for migration and electronic implementation paid greater attention to respondent usability and accessibility than previous iterations [11], there is much more to be done. Without consideration of the functional, sensory, physical and cognitive abilities of users of technologies in clinical trials, electronic data capture methods can easily be implemented in ways that cause difficulties for certain populations or exclude them altogether. We cannot take it for granted that digital tools increase diversity, especially in the absence of due consideration to accessibility.

## Methods

Each WCAG 2.2 success criteria, inclusive of mobile specific guidance, was compared by the authors to current ePROM best practices from Mowlem et al. [11], to identify areas of alignment or areas where there was conflict between the two guidances. Consideration for how accessibility criteria might affect ePROM implementation, as well as possible future areas of research to better understand the impact on measurement properties, is also provided. Given ePROM design best practices focus on the actual measure, its visual presentation, and ensuring the integrity and quality of the data captured, the criteria are considered from the perspective of how the measure is displayed, rather than the broader eCOA system (i.e., the app or website users log into to access the ePROMs). However, where certain criteria are relevant to the electronic system more widely but would not have a direct impact on the actual measure, comment is provided. Of note, the assumption is that the measure itself has been well-designed and is fit-for-purpose.

## Results

Table 1 lists the WCAG 2.2 accessibility success criteria that are relevant to screen-based ePROM data capture systems, compares them to current ePROM best practices, and provides considerations for incorporating accessibility success criteria into future ePROM best practices. Additional guidance relevant only to mobile devices is detailed in Table 2. Note, a classification level has not been allocated to any of the mobile specific guidance. For all WCAG 2.2 success criteria, including those that are not applicable to ePROMs, see Supplementary Material Table 1.

**Table 1:** WCAG 2.2 accessibility success criteria that are applicable to screen-based ePROM implementations, including if they are relevant to migration practices, the system user interface, or core functionality of the system, if the given success criteria are currently addressed in ePROM best practices, and the associated considerations for ePROM best practices

WCAG guideline & corresponding success criteria *		Category (Migration, Core system functionality, UI) **	Comment	Considerations for ePROM best practices
*1.1 Provide text alternatives for any non-text content so that it can be changed into other forms people need*,* such as large print*,* braille*,* speech*,* symbols or simpler language.*				
A	1.1.1 Non-text Content All non-text content that is presented to the user has a text alternative that serves the equivalent purpose, except for the situations listed below. **Controls**,** Input** If non-text content is a control or accepts user input, then it has a name that describes its purpose. (Refer to Success Criterion 4.1.2 for additional requirements for controls and content that accepts user input.) **Time-Based Media** If non-text content is time-based media, then text alternatives at least provide descriptive identification of the non-text content. (Refer to Guideline 1.2 for additional requirements for media.) **Test** If non-text content is a test or exercise that would be invalid if presented in text, then text alternatives at least provide descriptive identification of the non-text content. **Sensory** If non-text content is primarily intended to create a specific sensory experience, then text alternatives at least provide descriptive identification of the non-text content. **CAPTCHA** If the purpose of non-text content is to confirm that content is being accessed by a person rather than a computer, then text alternatives that identify and describe the purpose of the non-text content are provided, and alternative forms of CAPTCHA using output modes for different types of sensory perception are provided to accommodate different disabilities. **Decoration**,** Formatting**,** Invisible** If non-text content is pure decoration, is used only for visual formatting, or is not presented to users, then it is implemented in a way that it can be ignored by assistive technology.	Migration	This is not directly referred to in existing ePROM implementation best practices for screen-based devices, which focus on migration of the paper, typical text, format to electronic format. Where non-text content is part of a PROM, such as images, this content is migrated *as is* to ensure ‘faithful migration’ and maintenance of the original format and its measurement properties. Unless the copyright holder has specified a text alternative for this content that has been validated, then those implementing electronically would not be able to program a validated text alternative. A common response scale type, the Visual Analogue Scale (VAS), by its very definition (a line of fixed length, with text anchors at the extreme ends of the scale, but not words describing intermediate positions) would not allow for a text alternative.	For existing PROMs authors should create and validate text alternatives for non-text content that can then be provided when the measure is licensed for electronic implementation. For new PROMs, create text alternatives for images at the time of measure creation and do not include visual analogue scales as a response type.
*1.3 Create content that can be presented in different ways (for example simpler layout) without losing information or structure.*				
A	1.3.1 Info and Relationships Information, structure, and relationships conveyed through presentation can be programmatically determined or are available in text.	Migration, UI	This is not directly referred to in existing ePROM best practices but the core system functionality should support this.	The ePRO system should be able to support the programmatic determination of any information, structure, and relationships conveyed through visual presentation (e.g., user interface component labels are associated with the fields they are labeling, headings are tagged, lists can be identified with list markup).
A	1.3.2 Meaningful Sequence When the sequence in which content is presented affects its meaning, a correct reading sequence can be programmatically determined.	Core system functionality	An important part of PROM development and design includes the order of PRO questions and the order of the elements within an item. Therefore, the sequence in which the information is presented for a given PROM and its items is meaningful. Best practices to ensure a faithful migration involve maintaining this sequence, including response options being presented in the same order as the original format. Generally, a single item per screen is implemented as best practice to facilitate consistent visual presentation across modalities and device types. In some instances, multiple items per screen may be implemented, and this should follow the same meaningful sequence as on the paper format it is being migrated from.	The ePRO system should be able to support programming of the intended sequence of content when implementing an ePROM, to ensure the meaning of the content is maintained and the same for those using assistive technologies.
A	1.3.3 Sensory Characteristics Instructions provided for understanding and operating content do not rely solely on sensory characteristics of components such as shape, color, size, visual location, orientation, or sound.	Migration, UI	This is not directly referred to in ePROM best practices, but faithful migration involves no changes being made to the core wording of the measure (i.e., only implement minor changes to measure wording to align with the new mode of administration). If instructional text of the original measure refers to graphics, or uses only sensory characteristics for understanding content, then this would also be the case in the electronic implementation. As detailed in 1.1.1, unless the copyright holder has specified a text alternative for this content that has been validated, then those implementing electronically would not be able to program a validated text alternative.	For authors of existing PROMs, if their measures include instructional text that relies solely on sensory characteristics, they should consider creating and validating versions of their measures that provide additional information to clarify instructions that are dependent on this kind of information. For new PROMs, authors should ensure that they do not include content that relies solely on sensory characteristics. With regard to the eCOA system UI, any reference to components (e.g., navigation buttons, menus), should ensure that users do not have to rely solely on sensory characteristics of the components to understand and operate them. This should also be considered in any training modules.
AA	1.3.4 Orientation Content does not restrict its view and operation to a single display orientation, such as portrait or landscape, unless a specific display orientation is essential. Note Examples where a particular display orientation may be essential are a bank check, a piano application, slides for a projector or television, or virtual reality content where content is not necessarily restricted to landscape or portrait display orientation. Mobile specific Mobile application developers should try to support both orientations. If it is not possible to support both orientations, developers should ensure that it is easy for all users to change the orientation to return to a point at which their device orientation is supported. Changes in orientation must be programmatically exposed to ensure detection by assistive technology such as screen readers. For example, if a screen reader user is unaware that the orientation has changed the user might perform incorrect navigation commands.	Migration, Core system functionality	ePROM best practice is to keep the orientation consistent, and oftentimes ‘locked’, with the default being portrait orientation for mobile devices. Further, copyright holder guidelines on e-implementation (if available) often specify the orientation must be ‘locked’ to the dictated orientation for the given electronic mode. However, no evidence can be identified that claims content would only be understood in a certain orientation; whilst the aim of a consistently applied orientation is to facilitate standardization and reduce any potential frustration users may experience from orientation switching, it could lead to challenges for certain populations, such as those who have dexterity impairments and use a mounted device in a specific orientation.	Users should not be restricted to an enforced orientation and should be given the ability to switch to their preferred orientation. If this leads to the need for scrolling, then this should be implemented with the relevant functionality as outlined in Mowlem et al. [11]. Any changes in orientation must be programmatically exposed to ensure assistive technologies can detect it. Further research may be required to mitigate concerns that this could impact the measurement properties of the PROM.
AA	1.3.5 Identify Input Purpose The purpose of each input field collecting information about the user can be programmatically determined when: • The input field serves a purpose identified in the Input Purposes for user interface components section; and • The content is implemented using technologies with support for identifying the expected meaning for form input data.	UI	ePROMs do not collect the content types covered by this success criteria; however, ePRO systems should be secure and require a password to login, an input type that is covered.	N/A
*1.4 Make it easier for users to see and hear content including separating foreground from background.*				
A	1.4.1 Use of Color Color is not used as the only visual means of conveying information, indicating an action, prompting a response, or distinguishing a visual element. Note This success criterion addresses color perception specifically. Other forms of perception are covered in Guideline 1.3 including programmatic access to color and other visual presentation coding.	Migration, UI	This is not directly referred to in existing ePROM implementation best practices, but best practices focus on faithful migration of the paper format to electronic format, and if PROMs have been developed where color alone is used to convey information, they would be migrated in this way to maintain the measurement properties of the PROM. The eCOA provider has control over the colors used for the user interface, such as the navigation buttons.	For existing PROMs that include color as the only way to convey certain information, authors should create and validate versions where this is not the case. For new PROMs, authors should not use only color as a means to convey information. Within the UI, eCOA vendors should not only use color as a means to convey information.
AA	1.4.3 Contrast (Minimum) The visual presentation of text and images of text has a contrast ratio of at least 4.5:1, except for the following: **Large Text** Large-scale text and images of large-scale text have a contrast ratio of at least 3:1; **Incidental** Text or images of text that are part of an inactive user interface component, that are pure decoration, that are not visible to anyone, or that are part of a picture that contains significant other visual content, have no contrast requirement. **Logotypes** Text that is part of a logo or brand name has no contrast requirement. Mobile specific Mobile device content is viewed on smaller screens and in different conditions so this allowance for lessened contrast on large text must be considered carefully for mobile apps. For instance, the default point size for mobile platforms might be larger than the default point size used on non-mobile devices. When determining which contrast ratio to follow, developers should strive to make sure to apply the lessened contrast ratio only when text is roughly equivalent to 1.2 times bold or 1.5 times (120% bold or 150%) that of the default platform size. Note, however, that the use of text that is 1.5 times the default on mobile platforms does not imply that the text will be readable by a person with low vision. People with low vision will likely need and use additional platform level accessibility features and assistive technology such as increased text size and zoom features to access mobile content.	UI	This is not directly referred to in existing ePROM implementation best practices and should be applied to the entire UI of the system.	The ePRO system should ensure a contrast ratio of at least 4.5:1 for text and images of text, and 3:1 for large-scale text and images of large-scale-text. For mobile devices, the ePRO system should strive to have the contrast ratio set out in 1.4.6.
AA	1.4.4 Resize Text Except for captions and images of text, text can be resized without assistive technology up to 200% without loss of content or functionality. Mobile specific The following methods might be used: • Ensure that the browser pinch zoom is not blocked by the page’s viewport meta element so that it can be used to zoom the page to 200%. Restrictive values for user-scalable and maximum-scale attributes of this meta element should be avoided. Note: Relying on full viewport zooming (e.g. not blocking the browser’s pinch zoom feature) requires the user to pan horizontally as well as vertically. While this technique meets the success criteria it is less usable than supporting text resizing features that reflow content to the user’s chosen viewport size. It is best practice to use techniques that support text resizing without requiring horizontal panning. • Support for system fonts that follow platform level user preferences for text size. • Provide on-page controls A variety of methods allow the user to control content size on mobile devices with small screens. • OS-level features - Set default text size (typically controlled from the Display Settings) Note: System text size is often not supported by mobile browsers. - Magnify entire screen (typically controlled from the Accessibility Settings). Note: Using this setting requires the user to pan vertically and horizontally. - Magnifying lens view under user’s finger (typically controlled from the Accessibility Settings) • Browser-level features - Set default text size of text rendered in the browser’s viewport • Reading mode that renders main content at a user-specified text size - Magnify browser’s viewport (typically “pinch-zoom”). Note: Using this setting requires the user to pan vertically and horizontally. • Note: Some browsers have features that might modify this type of magnification (e.g. re-flowing the content at the new magnification level, overriding author attempts to prevent pinch-zoom).	Migration, Core system functionality, UI	Current ePROM best practice is to keep font size consistent within a single PROM and between PROMs on the same device where possible, though acknowledge that limitations in the size of the device may make it necessary to adjust the font size between individual measures or even screens within a PROM. Further, there should be consideration around not making text so small it is unreadable, and in this instance enable scrolling; however, this may still not satisfy requirements for some users. However, this is still under the control of the ePRO system, and established during the migration process, as opposed to allowing the user to alter the font size, which is generally not enabled. Some have even recommended disabling the ability for users to over-ride specific app settings, including font size [16]. Regulatory agencies, such as FDA, state that administrator or respondent burden will be reviewed to ensure quality and completeness of COA data, which includes font size that is too small to read easily, and encourages consideration around the use of large text for certain populations [23]. Smartphone devices tend to use pinch and zoom; however, this can lead to vertical and horizontal scrolling which may be problematic for usability and is not recommended in ePROM best practices.	Given that scrolling is recommended over making content so small it creates readability issues, enabling 200% text resizing that does not lead to loss of functionality or content, along with the appropriate scrolling functionality for the reflow, is recommended. It is recommended that research is conducted to understand the most optimal way to increase text size, including zooming and its impact on the measurement properties of a PROM, especially given not everyone will use assistive technologies. Early work on this topic has shown measurement comparability when allowing users to zoom [22] .
AAA	1.4.6 Contrast (Enhanced) The visual presentation of text and images of text has a contrast ratio of at least 7:1, except for the following: **Large Text** Large-scale text and images of large-scale text have a contrast ratio of at least 4.5:1; **Incidental** Text or images of text that are part of an inactive user interface component, that are pure decoration, that are not visible to anyone, or that are part of a picture that contains significant other visual content, have no contrast requirement. **Logotypes** Text that is part of a logo or brand name has no contrast requirement.	UI	As detailed in 1.4.3, this is not directly referred to in existing ePROM implementation best practices. Further, ePROMs do not use a large amount of variation within a single screen, and so this criteria is likely not deemed necessary (especially given in is AAA).	If possible, the ePRO system should meet this contrast criteria – especially for mobile devices - but 1.4.3 is sufficient.
AAA	1.4.8 Visual Presentation For the visual presentation of blocks of text, a mechanism is available to achieve the following: • Foreground and background colors can be selected by the user. • Width is no more than 80 characters or glyphs (40 if CJK). • Text is not justified (aligned to both the left and the right margins). • Line spacing (leading) is at least space-and-a-half within paragraphs, and paragraph spacing is at least 1.5 times larger than the line spacing. • Text can be resized without assistive technology up to 200% in a way that does not require the user to scroll horizontally to read a line of text on a full-screen window.	Migration, Core system functionality, UI	The ability for the foreground and background colour to be selected by the user is not something currently called out in ePROM best practices, and is considered part of the UI and not the PROM itself. Text alignment is not directly referred to in existing implementation best practices but migrations usually involve maintenance of the text alignment with the original format and copyright holder/license holder electronic implementation guidance often dictate this. Number of characters per line is also not detailed in best practices. There is not currently a specified ePROM implementation best practice for line spacing and it is often left to the vendor discretion or the copyright holder/license holder electronic implementation guidance may dictate this. Furthermore, line spacing, along with text/font size will be reduced if scrolling is not allowed. Current ePROM implementation best practice is to enable scrolling rather than reducing the font size to avoid causing reading difficulties. Resizing of text on a full-screen window refers to the most common sized desktop/laptop display with the viewport maximised and so this is not a requirement for mobile devices, which would use pinch and zoom.	Provide mechanisms to allow the user to: - select the foreground and background colours, to aid comprehension for those with certain visual or cognitive disabilities (this goes beyond dark mode). - enabling 200% text resizing for larger devices that does not lead to loss of functionality or content if zoom is not available; smartphone devices tend to use pinch and zoom (see 1.4.4) Do not implement PROMs using justified text alignment – conversation with copyright holders may be required if they dictate this, and where possible ensure the width of blocks of text is no more than 80 characters or glyphs. Where possible, have standardized line spacing in all ePROMs of at least space-and-a-half within paragraphs, and paragraph spacing is at least 1.5 times larger than the line spacing.
AA	1.4.10 Reflow Content can be presented without loss of information or functionality, and without requiring scrolling in two dimensions for: • Vertical scrolling content at a width equivalent to 320 CSS pixels; • Horizontal scrolling content at a height equivalent to 256 CSS pixels. Except for parts of the content which require two-dimensional layout for usage or meaning. Note 320 CSS pixels is equivalent to a starting viewport width of 1280 CSS pixels wide at 400% zoom. For web content which is designed to scroll horizontally (e.g., with vertical text), 256 CSS pixels is equivalent to a starting viewport height of 1024 CSS pixels at 400% zoom. Note Examples of content which requires two-dimensional layout are images required for understanding (such as maps and diagrams), video, games, presentations, data tables (not individual cells), and interfaces where it is necessary to keep toolbars in view while manipulating content. It is acceptable to provide two-dimensional scrolling for such parts of the content. Mobile specific The small screen size on many mobile devices limits the amount of content that can be displayed without scrolling. Positioning important page information so it is visible without requiring scrolling can assist users with low vision and users with cognitive impairments. If a user with low vision has the screen magnified only a small portion of the page might be viewable at a given time. Placing important elements before the page scroll allows those who use screen magnifiers to locate important information without having to scroll the view to move the magnified area. Placing important elements before the page scroll also makes it possible to locate content without performing an interaction. This assists users that have cognitive impairments such as short-term memory disabilities	Migration, Core system functionality	Current ePROM best practice is to make items self-contained when they are being implemented as a single item per screen, and to enable vertical scrolling as opposed to reducing font to a size that would cause reading difficulties, with the relevant scrolling functionality. Two-dimensional scrolling is not a recommended best practice. However, many copyright/license holders still do not allow vertical scrolling when implementing their measures electronically, and some sponsors also request no scrolling enabled. Placing important information before the page scroll may not be possible with ePROM, as items should be presented in the order of the original format.	Maintain existing best practices.
AA	1.4.11 Non-text Contrast The visual presentation of the following have a contrast ratio of at least 3:1 against adjacent color(s): **User Interface Components** Visual information required to identify user interface components and states, except for inactive components or where the appearance of the component is determined by the user agent and not modified by the author; **Graphical Objects** Parts of graphics required to understand the content, except when a particular presentation of graphics is essential to the information being conveyed.	Migration, UI	The contrast ratio used to identify user interface components and their states and graphics (e.g., measures that include body maps or faces), is not something that is documented in ePROM best practices. The identification of user interface components (e.g., control buttons) is part of the UI of the eCOA provider. The indication of state would not only apply to when a control has been selected, but also when a response option has been selected by the user.	Where ePROMs include graphics, and in instances where visual information is the only means to identify a control, or where visual information is used to indicate state, ensure a contrast ratio of 3:1 for the color of user interface components against adjacent colors.
AA	1.4.12 Text Spacing In content implemented using markup languages that support the following text style properties, no loss of content or functionality occurs by setting all of the following and by changing no other style property: • Line height (line spacing) to at least 1.5 times the font size; • Spacing following paragraphs to at least 2 times the font size; • Letter spacing (tracking) to at least 0.12 times the font size; • Word spacing to at least 0.16 times the font size. Exception: Human languages and scripts that do not make use of one or more of these text style properties in written text can conform using only the properties that exist for that combination of language and script.	Migration, UI	This is not directly referred to in existing ePROM implementation best practices and standards should be applied to the entire UI of the system. Some copyright holders/authors dictate certain spacing requirements or specify that the spacing of the electronic implementation must be matched with the paper format. However, this criterion is not about presenting it this way but ensuring that is the user is able to set the text spacing to those outlined, there is no loss of content or functionality. Previously, it has been suggested that eCOA systems should be set up to over-ride the ability for users to make changes ^16,20^.	If the user is able set the text spacing to those outlined ensure this is without loss of content or functionality.
AA	1.4.13 Content on Hover or Focus	N/A	This does not apply to ePROMs as they do not typically present additional content if a component receives focus.	N/A
*2.1 Make all functionality available from a keyboard.*				
A	2.1.1 Keyboard All functionality of the content is operable through a keyboard interface without requiring specific timings for individual keystrokes, except where the underlying function requires input that depends on the path of the user’s movement and not just the endpoints. Note This exception relates to the underlying function, not the input technique. For example, if using handwriting to enter text, the input technique (handwriting) requires path-dependent input but the underlying function (text input) does not. Note This does not forbid and should not discourage providing mouse input or other input methods in addition to keyboard operation.	UI	This is not detailed in ePROM best practices. Specific elements of ePROMs (e.g., response scale types) do not depend on the path of the user’s movement.	eCOA systems should allow for all functionality of content to be operable though a keyboard interface.
A	2.1.2 No Keyboard Trap If keyboard focus can be moved to a component of the page using a keyboard interface, then focus can be moved away from that component using only a keyboard interface, and, if it requires more than unmodified arrow or tab keys or other standard exit methods, the user is advised of the method for moving focus away. Note Since any content that does not meet this success criterion can interfere with a user’s ability to use the whole page, all content on the Web page (whether it is used to meet other success criteria or not) must meet this success criterion. See Conformance Requirement 5: Non-Interference.	UI	This is not detailed in ePROM best practices.	If 2.1.1 is applicable, then this must be possible. That is, eCOA systems should allow for all functionality of content to be operable though a keyboard interface, and should ensure there is no “keyboard trap” that prevents focus being moved away from the component using the keyboard.
*2.2 Provide users enough time to read and use content.*				
A	2.2.1 Timing Adjustable For each time limit that is set by the content, at least one of the following is true: **Turn off** The user is allowed to turn off the time limit before encountering it; or **Adjust** The user is allowed to adjust the time limit before encountering it over a wide range that is at least ten times the length of the default setting; or **Extend** The user is warned before time expires and given at least 20 s to extend the time limit with a simple action (for example, “press the space bar”), and the user is allowed to extend the time limit at least ten times; or **Real-time Exception** The time limit is a required part of a real-time event (for example, an auction), and no alternative to the time limit is possible; or **Essential Exception** The time limit is essential and extending it would invalidate the activity; or **20 h Exception** The time limit is longer than 20 h. Note This success criterion helps ensure that users can complete tasks without unexpected changes in content or context that are a result of a time limit. This success criterion should be considered in conjunction with Success Criterion 3.2.1, which puts limits on changes of content or context as a result of user action.	Core system functionality, UI	This is not detailed in ePROM best practices, but the time limit to complete a given ePROM is an important consideration requiring careful thought. A benefit of electronic implementation is the contemporaneousness of the data – having a time and date stamp associated with it, and the time window for completion of an ePROM is an essential part of the event, it should not be unlimited, and in many cases can require completion in a time window far less than 20 h. PROMs are typically time-bound – patients are rating themselves over a specified period of time, or within the current moment, and the time window for completion of an ePROM is an essential part of the event. Within the context of a clinical trial, this time window should not be unlimited, and in many cases study design requires completion in a time window far less than 20 h. Currently, there is no agreed upon best practice for the length of time a user is inactive before logout or how much time they are allowed to take to complete a given ePROM within the defined time window.	Given the importance of the contemporaneousness of ePROM data, time limits should not be turned off. Instead, it is recommended that the ePRO system informs/warns users if they are about to be logged out due to inactivity, and given 20 s to extend the time limit with an action (such as “tap the screen”), and that the user is allowed to extend the time limit at least 10 times, given that it remains within the allocated time window as defined by the protocol. Information on time limits for task completion and system logout due to inactivity should be included in participant training material.
AAA	2.2.4 Interruptions Interruptions can be postponed or suppressed by the user, except interruptions involving an emergency.	Core system functionality	This is not included in ePROM best practices. However, it is a concern for some sponsors with BYOD, as the user could be distracted by other notifications on their phone when completing an ePROM, though this criterion is detailed as being under the users control as opposed to the author of the solution.	Enable users to postpone any other notifications on their device when using the ePRO system, apart from alerts concerning emergencies.
AAA	2.2.6 Timeouts Users are warned of the duration of any user inactivity that could cause data loss, unless the data is preserved for more than 20 h when the user does not take any actions. Note Privacy regulations may require explicit user consent before user identification has been authenticated and before user data is preserved. In cases where the user is a minor, explicit consent may not be solicited in most jurisdictions, countries or regions. Consultation with privacy professionals and legal counsel is advised when considering data preservation as an approach to satisfy this success criterion.	Core system functionality	This is not detailed in ePROM best practices. In some systems, if a user exits an ePROM or logs out of the system prior to completion (i.e., have only partially completed a measure and not submitted the data), the data points that were completed would not be saved and the user would not be able to pick-up where they left off once they log back in. Whereas, in other systems, given they are still within the time window for completion of the measure as set out in the protocol and specified for the electronic build, the data entered thus far would be saved and they could pick up where they left off. This is something that requires further thought and discussion, as the user could be in a different ‘state’, leading to unreliable and conflicting data between the different time points at which the different parts of a measure were completed^6^. Further, multiple time stamps (and potentially dates) could be associated with the data which could cause analysis problems. With regard to accessibility, the important part for this criterion is that the user is warned of duration of any user inactivity that would lead to loss of the data they have entered so far.	Warn users of the duration of any user inactivity that would lead to loss of the data they have entered so far. As outlined in 2.2.1, this should be included in any participant training material. It is recommended that research is conducted on the impact of saving and completing later to the reliability of the data, and how much time should be allowed should this be a chosen option.
*2.4 Provide ways to help users navigate*,* find content*,* and determine where they are.*				
A	2.4.2 Page Titled Web pages have titles that describe topic or purpose.	UI	This is not detailed in ePROM best practices, and it not relevant to mobile apps, but would be relevant to web-based solutions.	Ensure the ePROM ‘page’ has a meaningful title specified in the TITLE element for web implementations.
A	2.4.3 Focus Order If a Web page can be navigated sequentially and the navigation sequences affect meaning or operation, focusable components receive focus in an order that preserves meaning and operability.	Core system functionality	An important part of PROM development and design includes the order of PRO questions and the order of the elements within an item. Therefore, the sequence in which the information is presented for a given PROM and its items is meaningful. Best practices to ensure a faithful migration involve maintaining this sequence, including response options being presented in the same order as the original format. Generally, a single item per screen is implemented as best practices to facilitate consistent visual presentation across modalities and device types. In some instances, multiple items per screen may be implemented, and this should follow the same meaningful sequence as on the paper format it is being migrated from.	The ePRO system should be programed to support logical movement (i.e., the intended meaningful sequence of content) through the focusable components using only the keyboard, to ensure the meaning of the content is maintained.
AA	2.4.5 Multiple Ways More than one way is available to locate a Web page within a set of Web pages except where the Web Page is the result of, or a step in, a process.	UI	This does not apply, as items within an ePROM should be completed within a specified order. It would be applicable to the ePRO system design as a whole, as this often contains a menu with other items, such as training, site contact details.	N/A
AA	2.4.6 Headings and Labels Headings and labels describe topic or purpose.	Migration	This does not apply exclusively to ePROMs but is relevant to the actual development of a COA which should be ‘fit-for-purpose’ in the target population. PROMS do not always have a title or heading that is meaningful to users. Response options would equate to labels in the electronic format, as they should describe the purpose of the associated field to the user, and again should have been developed to be ‘fit-for-purpose’.	N/A
AA	2.4.7 Focus Visible Any keyboard operable user interface has a mode of operation where the keyboard focus indicator is visible.	UI	This is not called out in ePROM best practices and applies to the UI of the system as a whole.	When an ePRO system is keyboard operable, there should be a mode of operation where the keyboard focus indicator is visible.
AA	2.4.11 Focus Not Obscured (Minimum) When a user interface component receives keyboard focus, the component is not entirely hidden due to author-created content. Note Where content in a configurable interface can be repositioned by the user, then only the initial positions of user-movable content are considered for testing and conformance of this Success Criterion. Note Content opened by the *user* may obscure the component receiving focus. If the user can reveal the focused component without advancing the keyboard focus, the component with focus is not considered visually hidden due to author-created content.	Core System Functionality, UI	This is not included in ePROM best practices, but if certain components are part of a measure, for example, if a date/time selection or dropdown menu was implemented, then when a user focuses on that component the content may go over the top of other content and obscure it. As they do not persist after being interacted with or once they are no longer the primary point of user interaction, they do not fail this criterion as they are not obscuring the new content with focus. Any modal dialog boxes that are implemented (such as an edit check or pop-up) would not be applicable as even when they appear directly on top of an item with focus, the dialog takes the focus and thus the new item with focus is visible and prevents interaction outside the modal until it is dismissed.	Ensure components that obscure other content do not persist after being interacted with or once they are no longer the primary point of user interaction, so they do not obscure other content that then receives focus.
AAA	2.4.12 Focus Not Obscured (Enhanced) When a user interface component receives keyboard focus, no part of the component is hidden by author-created content.	Core System Functionality, UI	See 2.4.11	See 2.4.11
AAA	2.4.13 Focus Appearance When the keyboard focus indicator is visible, an area of the focus indicator meets all the following: • is at least as large as the area of a 2 CSS pixel thick perimeter of the unfocused component or sub-component, and • has a contrast ratio of at least 3:1 between the same pixels in the focused and unfocused states. Exceptions: • The focus indicator is determined by the user agent and cannot be adjusted by the author, or • The focus indicator and the indicator’s background color are not modified by the author. Note What is perceived as the user interface component or sub-component (to determine the perimeter) depends on its visual presentation. The visual presentation includes the component’s visible content, border, and component-specific background. It does not include shadow and glow effects outside the component’s content, background, or border. Note Examples of sub-components that may receive a focus indicator are menu items in an opened drop-down menu, or focusable cells in a grid. Note Contrast calculations can be based on colors defined within the technology (such as HTML, CSS and SVG). Pixels modified by user agent resolution enhancements and anti-aliasing can be ignored.	UI	This is not called out in ePROM best practices and applies to the UI of the system as a whole.	In addition to 2.4.7 - when an ePRO system is keyboard operable, there should be a mode of operation where the keyboard focus indicator is visible – ensure the focus indicator is clearly visible and discernable.
*2.5 Make it easier for users to operate functionality through various inputs beyond keyboard.*				
A	2.5.2 Pointer Cancellation For functionality that can be operated using a single pointer, at least one of the following is true: **No Down-Event** The down-event of the pointer is not used to execute any part of the function; **Abort or Undo** Completion of the function is on the up-event, and a mechanism is available to abort the function before completion or to undo the function after completion; **Up Reversal** The up-event reverses any outcome of the preceding down-event; **Essential** Completing the function on the down-event is essential. Note Functions that emulate a keyboard or numeric keypad key press are considered essential. Note This requirement applies to web content that interprets pointer actions (i.e. this does not apply to actions that are required to operate the user agent or assistive technology). Mobile specific Activating elements via the mouse up or touch end event. Using the mouse up or touch end event to trigger actions helps prevent unintentional actions during touch and mouse interaction. Mouse users clicking on actionable elements (links, buttons, submit inputs) should have the opportunity to move the cursor outside the element to prevent the event from being triggered. This allows users to change their minds without being forced to commit to an action. In the same way, elements accessed via touch interaction should generally trigger an event (e.g. navigation, submits) only when the touch end event is fired (i.e. when all of the following are true: the user has lifted the finger off the screen, the last position of the finger is inside the actionable element, and the last position of the finger equals the position at touch start).	UI, Core System Functionality	This is not called out in ePROM best practices and applies to the UI of the system as a whole. It is also relevant to the core system functionality to support the implementation of ePROMs, as how a response option is selected and aborted or undone should be considered. In implementations where users are allowed to skip an item, it is important to consider when a user has selected a response option and then wants to “unselect” it so that they can skip the item. For response scale types such as the VRS and NRS, clicking/taping on the response option already selected could deselect it; however, for response scales types such as the VAS or a numeric entry, this method would not be likely to work for undoing their selection. A possible option would be to have a ‘reset screen’ button for users to return the item to its original presentation (i.e., with no response selected).	Ensure that all response scale types within an ePROM have a way for selection to be aborted or cancelled by the user.
A	2.5.3 Label in Name For user interface components with labels that include text or images of text, the name contains the text that is presented visually. Note A best practice is to have the text of the label at the start of the name.	Migration, UI	ePROM migration best practice is to ensure core wording is maintained, which would be applicable to response options and so the words which visibly ‘label’ the response type component would also be the word associated with the component programmatically. This should also be the case for any user interface components in the system as a whole that have labels that include text or images of text.	Maintain existing best practices.
AAA	2.5.5 Target Size (Enhanced) The size of the target for pointer inputs is at least 44 by 44 CSS pixels except when: **Equivalent** The target is available through an equivalent link or control on the same page that is at least 44 by 44 CSS pixels; **Inline** The target is in a sentence or block of text; **User Agent Control** The size of the target is determined by the user agent and is not modified by the author; **Essential** A particular presentation of the target is essential to the information being conveyed.	Migration, UI	See 2.5.8	See 2.5.8
AAA	2.5.6 Concurrent Input Mechanisms Web content does not restrict use of input modalities available on a platform except where the restriction is essential, required to ensure the security of the content, or required to respect user settings. Mobile specific Users can enter information on mobile devices in multiple ways such as on-screen keyboard, Bluetooth keyboard, touch, and speech.	UI	This is not detailed in ePROM best practices.	eCOA systems should allow for all content to be operable though different input mechanisms.
AA	2.5.7 Dragging Movements All functionality that uses a dragging movement for operation can be achieved by a single pointer without dragging, unless dragging is essential or the functionality is determined by the user agent and not modified by the author. Note This requirement applies to web content that interprets pointer actions (i.e. this does not apply to actions that are required to operate the user agent or assistive technology).	Core System Functionality	Current ePROM best practice for interacting with, and operating the VAS is to allow the user to select a point on the line (their response) by either tapping or sliding (or both), and so meets this criterion as both options to operate the response scale are possible.	Maintain existing best practices.
AA	2.5.8 Target Size (Minimum) The size of the target for pointer inputs is at least 24 by 24 CSS pixels, except where: • **Spacing**: Undersized targets (those less than 24 by 24 CSS pixels) are positioned so that if a 24 CSS pixel diameter circle is centered on the bounding box of each, the circles do not intersect another target or the circle for another undersized target; • **Equivalent**: The function can be achieved through a different control on the same page that meets this criterion; • **Inline**: The target is in a sentence or its size is otherwise constrained by the line-height of non-target text; • **User agent control**: The size of the target is determined by the user agent and is not modified by the author; • **Essential**: A particular presentation of the target is essential or is legally required for the information being conveyed. Note Targets that allow for values to be selected spatially based on position within the target are considered one target for the purpose of the success criterion. Examples include sliders, color pickers displaying a gradient of colors, or editable areas where you position the cursor. Note For inline targets the line-height should be interpreted as perpendicular to the flow of text. For example, in a language displayed vertically, the line-height would be horizontal. Mobile specific The high resolution of mobile devices means that many interactive elements can be shown together on a small screen. But these elements must be big enough and have enough distance from each other so that users can safely target them by touch. Best practices for touch target size include the following: • Ensuring that touch targets are at least 9 mm high by 9 mm wide. • Ensuring that touch targets close to the minimum size are surrounded by a small amount of inactive space. Note: This size is not dependent on the screen size, device or resolution. Screen magnification should not need to be used to obtain this size, because magnifying the screen often introduces the need to pan horizontally as well as vertically, which can decrease usability.	Migration, UI	Current ePROM best practices are that the entire NRS must be visible on one screen without scrolling, that each number response option is the same size, and the NRS does not wrap across lines. Further, on mobile devices it is common to lock the orientation to portrait (as detailed in 1.3.4). Given that changing this would essentially change the information or functionality of the content, the criterion details that the target does not need to meet the minimum size detailed as the requirements are essential to the scale type. This is likely to be especially applicable to mobile devices where screen sizes are small.	eCOA systems should ensure that all interface components have a target size of 24 by 24 CSS pixels – unless a smaller size is essential to the information being conveyed. Specifically for mobile devices, it is recommended that touch targets are at least 9 mm high by 9 mm wide, and that those close to the minimum size are surrounded by a small amount of inactive space. In instances where the above are not possible, consider the use of devices with larger screen sizes or enabling orientation changes (see 1.3.4.). It is recommended that future research is conducted to evaluate different presentations of the NRS (e.g., a vertical presentation), that could optimize usability and accessibility, to ensure measurement comparability is maintained.
*3.1 Make text content readable and understandable.*				
A	3.1.1 Language of Page The default human language of each Web page can be programmatically determined.	UI	When ePROMs are used in global studies, they will be translated into the specific locales required and the correct locale will be assigned to each user on the device to ensure they receive the localisation of the ePROM appropriate to them. The platform should also be set-up so the language can be programmatically identified by assistive technologies.	Ensure the ePRO system enables the programmatic identification of a page’s language.
*3.2 Make Web pages appear and operate in predictable ways.*				
A	3.2.1 On Focus When any user interface component receives focus, it does not initiate a change of context.	Core system functionality	Current ePROM best practice is that there should be no auto-advance to the next screen on selection of a response option, without the respondent selecting the applicable navigation button.	Maintain existing best practices.
A	3.2.2 On Input Changing the setting of any user interface component does not automatically cause a change of context unless the user has been advised of the behavior before using the component.	Core system functionality	Current ePROM best practices is that there should be no auto-advance to the next screen on selection of a response option, without the respondent selecting the applicable navigation button, and that there should be a final save and submit screen that informs users when they have reached the end of a measure and can go back and review or change any responses before saving and submitting, at which point they will no longer be able to.	Maintain existing best practices.
AA	3.2.3 Consistent Navigation Navigational mechanisms that are repeated on multiple Web pages within a set of Web pages occur in the same relative order each time they are repeated, unless a change is initiated by the user.	UI	Current ePROM best practice is that navigation buttons should be placed consistently on each screen.	Maintain existing best practices.
AA	3.2.4 Consistent Identification Components that have the same functionality within a set of Web pages are identified consistently. Mobile specific Elements that trigger changes should be sufficiently distinct to be clearly distinguishable from non-actionable elements (content, status information, etc.). Providing a clear indication that elements are actionable is relevant for web and native mobile applications that have actionable elements like buttons or links, especially in interaction modes where actionable elements are commonly detected visually (touch and mouse use). Interactive elements must also be detectable by users who rely on a programmatically determined accessible name (e.g. screen reader users). Visual users who interact with content using touch or visual cursors (e.g. mice, touchpads, joysticks) should be able to clearly distinguish actionable elements such as links or buttons. Existing interface design conventions are aimed at indicating that these visual elements are actionable. The principle of redundant coding ensures that elements are indicated as actionable by more than one distinguishing visual feature. Following these conventions benefits all users, but especially users with vision impairments. Visual features that can set an actionable element apart include shape, color, style, positioning, text label for an action, and conventional iconography. Examples of distinguishing features: 1. Conventional shape: Button shape (rounded corners, drop shadows), checkbox, select rectangle with arrow pointing downwards 2. Iconography: conventional visual icons (question mark, home icon, burger icon for menu, floppy disk for save, back arrow, etc.) 3. Color offset: shape with different background color to distinguish the element from the page background, different text color 4. Conventional style: Underlined text for links, color for links Conventional positioning: Commonly used position such as a top left position for back button (iOS), position of menu items within left-aligned lists in drop-down menus for navigation.	UI	This is not detailed in ePROM best practices, but assumed to be part of the UI. This can be challenging when sometimes copyright holders dictate how they would like the buttons to be labelled; for example, some want a “Next” button on the first screen and others want a “Start” button which could lead to different navigation button names across measures within a study. If vendors are using operating system native components, this could also lead to inconsistent identification, as IOS and Android sometimes use different labels for navigation buttons. With the increase in BYOD and the potential that a user could swap to a different operating system within a study (e.g., if they get a new device), this could be problematic and cause confusion, in addition to if web-back ups are used that also have different identification.	Use consistent identification and labeling of functional components that appear repeatedly throughout the eCOA system. If icons or other non-text items also have the same functionality, these should be consistent as well as their text alternatives. It can be argued that the functional components are part of the eCOA vendors product, and not the actual measure, and so copyright holders should not dictate this identification, especially given the confusion this might cause for users if multiple measures use different identifications.
AAA	3.2.5 Change on Request Changes of context are initiated only by user request or a mechanism is available to turn off such changes.	Core system functionality	Current ePROM best practices are that there should be no auto-advance to the next screen on selection of a response option, without the respondent selecting the applicable navigation button, and that there should be a final save and submit screen that informs users when they have reached the end of the measure that they can go back and review or change any responses before saving and submitting, at which point they will no longer be able to and the user will control if they go back or save and submit though their navigation selection.	Maintain existing best practices.
A	3.2.6 Consistent Help If a Web page contains any of the following help mechanisms, and those mechanisms are repeated on multiple Web pages within a set of Web pages, they occur in the same order relative to other page content, unless a change is initiated by the user: • Human contact details; • Human contact mechanism; • Self-help option; • A fully automated contact mechanism. Note Help mechanisms may be provided directly on the page, or may be provided via a direct link to a different page containing the information. Note For this Success Criterion, “the same order relative to other page content” can be thought of as how the content is ordered when the page is serialized. The visual position of a help mechanism is likely to be consistent across pages for the same page variation (e.g., CSS break-point). The user can initiate a change, such as changing the page’s zoom or orientation, which may trigger a different page variation. This criterion is concerned with relative order across pages displayed in the same page variation (e.g., same zoom level and orientation).	Core System Functionality	ePROMs do not include the content types covered by this success criteria; however, ePRO systems should provide contact details for users to access help and so should be mindful of this.	N/A
*3.3 Help users avoid and correct mistakes.*				
A	3.3.1 Error Identification If an input error is automatically detected, the item that is in error is identified and the error is described to the user in text.	Core system functionality	Current ePROM best practice is to prevent out-of-range responses which can be programmed by limiting out of range data and automatically detecting when this occurs by the user, and informing the user of an error. Detail regarding the recommended behavior to communicate the out-of-range or illogical response is not provided (e.g., how users are informed that their response was out-of-range and therefore not accepted by the system), and would depend on the content type (e.g., are they simply told it is out of range or should detail of the range be provided? ).	Maintain existing best practices, and expand with recommendation on how detail of the error should be communicated to users (i.e., provide the available programmed range, or just inform the user of an error and how to action it).
A	3.3.2 Labels or Instructions Labels or instructions are provided when content requires user input. Mobile specific The ability to provide control via custom touchscreen and device manipulation gestures can help developers create efficient new interfaces. However, for many people, custom gestures can be a challenge to discover, perform and remember. Therefore, instructions (e.g. overlays, tooltips, tutorials, etc.) should be provided to explain what gestures can be used to control a given interface and whether there are alternatives. To be effective, the instructions should, themselves, be easily discoverable and accessible. The instructions should also be available anytime the user needs them, not just on first use, though on first use they may be made more apparent through highlighting or some other mechanism. On some mobile devices, the standard keyboard can be customized in the device settings and additional custom keyboards can be installed. Some mobile devices also provide different virtual keyboards depending on the type of data entry. This can be set by the user or can be set to a specific keyboard. For example, using the different HTML5 form field controls (see Method Editor API) on a website will show different keyboards automatically when users are entering in information into that field. Setting the type of keyboard helps prevent errors and ensures formats are correct but can be confusing for people who are using a screen reader when there are subtle changes in the keyboard.	Migration, UI	Current ePROM best practice to ensure a faithful migration means that there should not be any changes to the measure wording (apart from those to align with the new mode of administration) and that all information required to fully understand an item and select the desired response must be included. This would include each response option in the electronic format having an appropriate label so they know what they are selecting – i.e., any option for user input provided to a user should have a label. Providing on-device instruction and training on system use is also a best practice and should include sample questions composed of the input types they will encounter during the study.	Maintain existing best practices, and expand to include recommendation that when a virtual keyboard is used, the keyboard presented for a given item is relevant to the response format (e.g., present a number pad for numeric entry).
AA	3.3.3 Error Suggestion If an input error is automatically detected and suggestions for correction are known, then the suggestions are provided to the user, unless it would jeopardize the security or purpose of the content.	Core System Functionality	As detailed in 3.3.1, current ePROM best practice is to prevent out-of-range responses which can be programmed by limiting out of range data and automatically detecting when this occurs from the user; in this case, the user may be informed of the range but not a more specific suggestion as this could jeopardize the purpose of the content (i.e. collecting accurate self-reports from the participant).	N/A
AAA	3.3.5 Help Context-sensitive help is available.	Core system functionality, UI	Providing training on system use is an ePROM best practice, and assistance with understanding of the content of the PROM itself is relevant to the actual development of the measure by the author, which should be ‘fit-for-purpose’ in the target population.^5^ This criterion details that context-sensitive help only needs to be provided when a label is not sufficient to describe the functionality, and so any UI components (e.g., “Next” button) should have labels that achieve this.	Maintain existing best practices.
AAA	3.3.6 Error Prevention (All) For Web pages that require the user to submit information, at least one of the following is true: **Reversible** Submissions are reversible. **Checked** Data entered by the user is checked for input errors and the user is provided an opportunity to correct them. **Confirmed** A mechanism is available for reviewing, confirming, and correcting information before finalizing the submission.	Migration, Core system functionality	Current ePROM best practice is to include a final save and submit screen that informs respondents when they have reached the end of an ePROM and that they can go back and review and change any responses before submitting them, providing the opportunity to prevent errors. After the data is submitted, an error may still be corrected by contacting site personnel. ePROM best practices also include capturing a data point for all items - this means if a respondent is allowed to skip that an edit-check is triggered to confirm their intention to skip the question without providing a response. ePROM best practices also include the use of edit-checks for out-of-range or illogical responses by programming a pop-up message when such an entry occurs. Both best practices check for input errors and provide the opportunity for the user to correct them.	Maintain existing best practices.
AA	3.3.8 Accessible Authentication (Minimum) A cognitive function test (such as remembering a password or solving a puzzle) is not required for any step in an authentication process unless that step provides at least one of the following: **Alternative** Another authentication method that does not rely on a cognitive function test. **Mechanism** A mechanism is available to assist the user in completing the cognitive function test. **Object Recognition** The cognitive function test is to recognize objects. **Personal Content** The cognitive function test is to identify non-text content the user provided to the Web site. Note “Object recognition” and “Personal content” may be represented by images, video, or audio. Note Examples of mechanisms that satisfy this criterion include: 1. support for password entry by password managers to reduce memory need, and 2. copy and paste to reduce the cognitive burden of re-typing.	Core system functionality	This criterion is not relevant to the ePROM itself; however, ePRO systems should be secure and require a password to login, an input type that is covered, and so should be mindful of this.	N/A
*4.1 Maximize compatibility with current and future user agents*,* including assistive technologies.*				
A	4.1.2 Name, Role, Value For all user interface components (including but not limited to: form elements, links and components generated by scripts), the name and role can be programmatically determined; states, properties, and values that can be set by the user can be programmatically set; and notification of changes to these items is available to user agents, including assistive technologies. Note This success criterion is primarily for Web authors who develop or script their own user interface components. For example, standard HTML controls already meet this success criterion when used according to specification.	UI, Core system functionality	This is not directly referred to in existing ePROM best practices but the core system functionality should support this. However, detail is provided, that when navigating between screens that have already been encountered and a response option selected, then this previous response selection should be clear to the user, including identification by assistive technologies. Again, this would be challenging for the VAS given the selection is a visual point, and the response not associated with an identifiable label to the user.	The ePRO system should be able to support the programmatic determination of the name and role of user interface components, and any changes to the state of these elements should be identifiable by all. For new PROMs, do not include visual analogue scales as a response type.
AA	4.1.3 Status Messages In content implemented using markup languages, status messages can be programmatically determined through role or properties such that they can be presented to the user by assistive technologies without receiving focus.	UI, Core system functionality	Current ePROM best practices are to capture a data point for all items. If a respondent is not allowed to skip an item, a pop-up message should be programmed to appear if they press the “Next” button without selecting a response, indicating the requirement to provide a response before being able to proceed. If respondents are allowed to skip an item, then an edit-check should be triggered to confirm their intention so skip the question without providing a response. In both these scenarios, these would not be classified as a status message as the message is displayed in a dialog that takes focus and this change in context would be announced by the screen reader. Another ePROM best practice is to prevent out-of-range or illogical responses by programming a pop-up message. As above this would not classify as a status message. If these functionalities were to be implemented, not as a pop-up displayed in a dialog and a change in context, but by adding this new information on the actual page itself which is not necessarily given focus, then this success criteria would be relevant.	Maintain existing best practices. If messages displayed to the user are not going to be implemented in a dialog but instead by adding new information to the page itself, then the ePRO system should ensure that they can be programmatically determined and presented to users of assistive technologies without receiving focus.

* A, AA, and AAA refer to the three levels of conformance representing a different level of accessibility, with AAA being the greatest level off accessibility, and each success criteria have an appliable level of conformance assigned based on satisfaction of the success criteria (i.e., meeting the requirements). To meet AA compliance, both A and AA success criteria must be met, and to meet AAA compliance, A, AA, and AAA success criteria must be met. Of note, if there is no content to which a success criterion applies, then that success criterion is deemed to be satisfied. Further details can be found on the WAI website [7].

** Migration = the success criteria are directly related to best practices for the migration from paper and electronic implementation of a PROM; Core system functionality = the success criteria are related to functionality the system should support to enable the electronic implementation of PROMS; UI = the success criteria are related to the ePRO provider’s platform as a whole, rather than solely to the ePROM and should be created as a platform standard; N/A = Not Applicable to ePROMs

Current ePROM best practices refers to Mowlem et al. [11]

**Table 2:** Mobile specific accessibility guidance from W3C, including if they are relevant to migration practices, the system user interface, or core functionality of the system, if the given success criteria are currently addressed in ePROM best practices, and the associated considerations for ePROM best practices

Mobile specific guidance	Category (Migration, Core system functionality, UI) *	Comment	Considerations for ePROM best practices
**Small Screen Size** Some best practices for helping users to make the most of small screens include • Minimizing the amount of information that is put on each page compared to desktop/laptop versions by providing a dedicated mobile version or a responsive design: - a dedicated mobile version contains content tailored for mobile use. For example, the content may contain fewer content modules, fewer images, or focus on important mobile usage scenarios. o a responsive design contains content that stays the same, but CSS stylesheets are used to render it differently depending on the viewport width. For example, on narrow screens the navigation menus may be hidden until the user taps a menu button. - Providing a reasonable default size for content and touch controls (see “B.2 Touch Target Size and Spacing”) to minimize the need to zoom in and out for users with low vision. • Positioning form fields below, rather than beside, their labels (in portrait layout)	Migration, UI	Much of ePROM design and implementation best practice is focused on standardizing presentation for all users across all device types (including different screen sizes) to reduce the risk of any variation in the way participants respond. Content included in an ePROM is essential to it, and this should not differ as a function of the device type, and so dedicated mobile versions that contains less content is not applicable for ePROMs. As detailed, ePROM best practices are to enable scrolling over disabling it and having a smaller text size that could cause readability issues, and so the need for pinch and zoom should be minimized in these instances.	Maintain existing ePROM best practices, in addition to those outlined in Table 1.
**Placing buttons where they are easy to access** Mobile sites and applications should position interactive elements where they can be easily reached when the device is held in different positions. When designing mobile web content and applications many developers attempt to optimize use with one hand. This can benefit people with disabilities who may only have one hand available, however, developers should also consider that an easy-to-use button placement for some users might cause difficulties for others (e.g. left- vs. right-handed use, assumptions about thumb range of motion). Therefore, flexible use should always be the goal. Some (but not all) mobile operating systems provide work-around features that let the user temporarily shift the display downwards or sideways to facilitate one-handed operation.	UI	This is not detailed in ePROM best practices. Consistent navigation is detailed, but not ease of access of placement. In ePROMs, button placement has traditionally been focused on ensuring users are able to see an item and all its response options in a single glance, and minimizing the risk of a user navigating away from the screen before having seen all content.	Implement interactive elements, such as navigation buttons or response option buttons for the VRS, so that they span the entire width of the screen where possible; that is, they are not just on one side (as in Fig. 2.), where this might work for some and not others (e.g., left- vs. right-handed users) but one above the other.
**Provide easy methods for data entry** Text entry can be time-consuming and difficult in certain circumstances. Reduce the amount of text entry needed by providing select menus, radio buttons, check boxes or by automatically entering known information (e.g. date, time, location).	UI	Current ePROM best practice to ensure a faithful migration means that there should not be any changes to the measure wording (apart from those to align with the new mode of administration). However, free text is not advised when implementing PROMs electronically. Items with free-text responses in PROMs are not usually used for scoring and can introduce the risk that the data may be challenging to interpret, code, and manage. An alternative is providing a prepopulated list of common responses to the item. Best practices also include that when a respondent lands on a screen, a response should not be preselected but that for date/time entry, the balance between usability and not prompting a particular response requires consideration on a case-by-case basis; for example, providing the current date/time may aid usability for respondents reporting events in real time, despite the original format not providing this.	Maintain existing best practices.

* Migration = the success criteria are directly related to best practices for the migration from paper and electronic implementation of a PROM; Core system functionality = the success criteria are related to functionality the system should support to enable the electronic implementation of PROMS; UI = the success criteria are related to the ePRO provider’s platform as a whole, rather than solely to the ePROM and should be created as a platform standard; N/A = Not Applicable to ePROMs

Current ePROM best practices refers to Mowlem et al. [11]

**Fig. 2:**
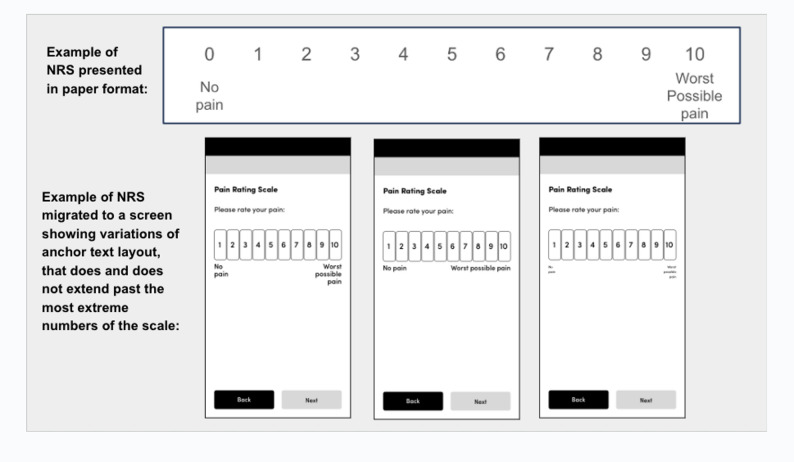
Illustration of a numeric rating scale (NRS) in a paper format that has been migrated to electronic format with differing anchor text size and text wrapping

Many of the accessibility criteria address programming of the software to ensure content is implemented in a way that enhances overall accessibility and can be reliably interpreted by assistive technologies (e.g., screen readers, magnifiers), by accounting for them at the point of software development. Table 3 lists the accessibility practices that could be applied to eCOA systems now without concern for the integrity of the measure, given their focus is not on changing the measure content or the mode of administration, but the software being programmed and built in a way that enhances the overall accessibility and its usability with assistive technologies. Consistent testing of the system for compatibility with assistive technologies is encouraged, and the impact of the actual use of assistive technologies on measure integrity should be explored further.

**Table 3:** Recommended ePROM accessibility best practices that can be adopted now within the context of existing ePROM implementation best practices

	Recommendation
1.	Support the programmatic determination of any information, structure, and relationships conveyed through visual presentation (e.g., UI component labels are associated with the fields they are labeling, headings are tagged, lists can be identified with list markup).
2.	Support programming of the intended sequence of content when implementing an ePROM to ensure the meaning of the content is maintained and the same for those using assistive technologies.
3.	Support the programmatic determination of the name and role of user interface components, and any changes to the state of these elements should be identifiable by all.
4.	If messages displayed to the user are not going to be implemented in a dialog but instead by adding new information to the page itself, ensure that they can be programmatically determined and presented to users of assistive technologies without receiving focus.
5.	Ensure that all interface components - where not essential to the information being conveyed - have a target size of 24 by 24 CSS pixels. - Specifically for mobile devices, it is recommended that touch targets are at least 9 mm high by 9 mm wide, and that those close to the minimum size are surrounded by a small amount of inactive space. In instances where the above are not possible, consider the use of devices with larger screen sizes or enabling orientation changes.
6.	Enable the programmatic identification of a page’s language.
7.	Use consistent identification and labeling of functional components that appear repeatedly throughout the eCOA system. If icons or other non-text items also have the same functionality, these should be consistent as well as their text alternatives.
8.	Allow all content to be operable though different input mechanisms.
9.	Allow for all functionality of content to be operable though a keyboard interface and: - ensure there is no “keyboard trap” that prevents focus being moved away from the component using the keyboard - support a mode of operation where the keyboard focus indicator is visible and discernable - support logical movement (i.e., the intended meaningful sequence of content) through the focusable components using only the keyboard, to ensure the meaning of the content is maintained.
10.	When a virtual keyboard is used, ensure the keyboard presented for a given item is relevant to the response format (e.g., present a number pad for numeric entry).
11.	Ensure the ePROM ‘page’ has a meaningful title specified in the TITLE element for web implementations.
12.	Ensure components that obscure other content do not persist after being interacted with or when no longer the primary point of user interaction, so they do not obscure other content that then receive focus.
13.	Ensure a contrast ratio of at least 4.5:1 for text and images of text, and 3:1 for large-scale text and images of large-scale-text. For mobile devices, the ePRO system should strive the contrast ratio 7:1 and 4.5:1, respectively.
14.	For graphics and instances where visual information is the only means to identify a control, or where visual information is used to indicate state, ensure a contrast ratio of 3:1 for the color of user interface components against adjacent colors.
15.	Do not use justified text alignment, and where possible ensure the width of blocks of text is no more than 80 characters or glyphs.
16.	Do not solely use sensory characteristics for the understanding and operation of components.
17.	Do not use color as the only means to convey information.
18.	Inform/warn users if they are about to be logged out due to inactivity and if this could cause any data loss, provide 20 s for them to extend the time limit with an action (such as “tap the screen”), and allow them to extend the time limit at least 10 times, given that it remains within the allocated time window as defined by the protocol.
19.	Ensure that all response scale types within an ePROM have a way for selection to be aborted or cancelled by the user.
20.	Where possible, have standardized line spacing of at least space-and-a-half within paragraphs, and paragraph spacing of at least 1.5 times more than the line spacing.
21.	If the user is able to set text spacing (increase in spacing between lines, words, letters, and paragraphs) ensure this is without loss of content or functionality.
22.	Do not enforce device orientation and provide the ability for users to switch orientation of the device. Any changes in orientation must be programmatically exposed to ensure assistive technologies can detect it.
23.	Allow the user to select foreground and background colors (beyond dark/light mode).
24.	If zoom is not available, enable 200% text resizing that does not lead to loss of functionality or content, along with the appropriate scrolling functionality for the reflow.
25.	Enable users to postpone any other notifications on their device when using the ePRO system, apart from alerts concerning emergencies.

Beyond considerations for assistive technologies, the main tensions between accessibility success criteria and ePROM best practices identified in this evaluation concerned the ability to increase content size and device orientation.

### Increasing the size of content

Current ePROM best practice is to enable vertical scrolling (horizontal is not recommended) instead of reducing text to a small enough size that it could cause reading difficulties. However, some still caution against the use of scrolling when implementing ePROMs [20] and some measure copyright holders do not allow scrolling. Further, the amount of scrolling required for a given item tends to be programmed as the same for all users (i.e., the text size is still dictated by the system as opposed to allowing the user to alter it or presenting content at the size set on the device), and so may still not satisfy the text size required by some users. Some have even recommended, with best intentions regarding the maintenance of measurement properties, that in BYOD scenarios the user should not be able to over-ride specific app display settings [20], including font size [16]. The use of zoom has not yet been addressed in ePROM best practices and has only recently received attention in research [22], with preliminary findings showing measurement comparability.

In contrast, WCAG 2.2 success criteria indicate that it should be possible for users to increase text size up to 200% without the loss of content or functionality; that is, it reflows (wraps) with scrolling in one dimension (i.e., vertically). Given the reducing concern around scrolling on the impact of an ePROMs measurement properties [11, 20], and upcoming work extending this to zoom functionality [22], it is worth considering if 200% text resizing or zooming should be possible for users, combined with the existing recommendations around how to implement scrolling functionality outlined in Mowlem et al. [11] Based on accessibility guidelines, it will be important that zooming does not lead to scrolling in two dimensions (i.e., vertically and horizontally). It is also recommended that further research on the impact of zooming and text resizing on the measurement properties of an ePROM is conducted, including understanding the most optimal method based on user preference. This should include the full item, inclusive of the response options for all response scale types (verbal rating scale, numeric rating scale, visual analogue scale), and not focus only on increasing the size of the item stem (i.e., the question text or statement to be rated).

### Orientation

Current ePROM best practice is to keep the device orientation consistent, along with recommending that ePROMs are presented in portrait on smartphones, and that respondents should not be able to manually alter the orientation (e.g., through rotation of the device). Of note, this is not referring to changing the orientation of the response scale types themselves (e.g., an NRS being presented vertically rather than the traditional horizontal). In contrast, WCAG 2.2 success criteria states that users should be allowed to switch the orientation of their device between portrait and landscape as they wish, unless a certain orientation is deemed essential to the content.

To date, there has been no published evidence that changing device orientation - or using one orientation over the other - would impact the measurement properties of the PROM. Therefore, it is difficult to argue that a given orientation is essential to the content being presented. The intent of the ePROM best practice recommending against allowing users to switch orientation is to facilitate standardization of the presentation of measures across respondents and eliminate the risk or formatting issues tied to changing orientation. However certain populations, such as those who have dexterity impairments, may benefit from a mounted device in a specific orientation, and so this ePROM best practice should be reconsidered. As system providers develop their software, the ability of content to function in either orientation and allowing users to switch to their preferred orientation, as well as the programmatic exposing of the orientation to assistive technologies, should be included and tested adequately. Conducting research on the measurement comparability between orientations could also help mitigate any concerns that the integrity of the measure could be impacted.

### Recommendations for further research

In addition to the areas identified above (the impacts of assistive technology, orientation, zooming, and text resizing), it is recommended that the following are the subject of future studies to enable a clear understanding on whether meeting the accessibility criteria would impact the measurement properties of the PROM:

- Horizontal vs. vertical presentations of the NRS (this could extend to the VAS, but given this is not a recommended response type for an accessible measure, the NRS should be the focus) (related to 2.5.8 Target Size [Minimum], see Table 1)
- The impact of saving partially completed measures and completing them later on the reliability of the data, and potential time limits for this (related to 2.2.6 Timeouts, see Table 1).

### Implications for measure development

As mentioned, the focus of this evaluation was on the implementation of the PROM in an electronic system, under the assumption that the measure itself has been well-designed and is fit-for-purpose. If the PROM itself (regardless of mode of administration) has not been developed in an accessible way, then this fundamental lack of accessibility is unlikely be resolved through electronic implementation (e.g., the VAS will be inherently difficult for vision-impaired individuals to complete in any mode). That said, the following are recommended to make existing PROMs accessible and ensure the accessibility of new measures.

For existing measures:

- Create and validate text alternatives for non-text content (e.g., image-based response options) that can be provided when the measure is licensed for electronic implementation
- If instructional text relies solely on sensory characteristics, create and validate versions that provide additional information to clarify instructions dependent on this kind of information
- If color is the only way to convey certain information, create and validate versions where this is not the case

For new measures:

- Create text alternatives for images at the time of measure creation
- Do not use the VAS as a response type
- Do not use content that relies solely on sensory characteristics
- Do not use color as the only means to convey information

## Discussion

To date in the field of electronic capture of PROMs in clinical trials, there has been greater focus on the comparability of the electronic implementation to the paper format rather than to the general accessibility of these technology driven solutions. Best practices for ePROMs have largely been developed by individuals in the field of outcomes measurement and eCOA research who have different disciplinary priorities compared to those working in more general web- and app-based solutions, resulting in tensions between ePROM design best practices focused on maintaining measurement properties, and general web- and app-design best practices to provide solutions that are accessible to all. The clinical trial industry prides itself on being “patient-centric” yet has been slow in adopting relatively simple and widespread technologic solutions that can address common challenges for users of technologies. Recent ePROM best practices have brought stronger consideration to usability and accessibility but further progress is needed, with much to learn from the world of good web and app design.

This article has outlined that many accessibility best practices can be incorporated into ePROM implementations *today*, most of which are focused on the programming of the software and criteria that will not alter the content of a PROM - it is urged that those who develop these data capture systems adopt these wherever possible. Recommendations for further research that may be required to support accessible approaches without impacting the measurement properties of the PROM were also outlined.

The focus here was specifically on the accessibility of the ePROM implementation as opposed to the eCOA system more widely, given this is what is addressed by current ePROM best practices [11]. However, from the user perspective it is unlikely that they differentiate their interaction between the implementation of the actual measure and the wider system, such as the app or website users access the ePROM through. Whether this separation is a logical one should be considered when developing future best practices.

Further, the actual use of assistive technologies for accessing and completing ePROMs and how this may impact measurement comparability was beyond the scope of this paper, but requires further thought and discussion; for example, understanding if completion of a measure by those using a screen-reader versus those who are not leads to measurement non-comparability. ePROM best practices have been largely focused on reducing variability between modes of administration, and so allowing users to engage with the content of a measure via an assistive technology, for example a screen reader, is a significant change in thinking for the industry. However, it is worth noting that from the earliest days of ePRO we have been displaying questions and response options on a single, relatively small screen, which is already a significant visual departure from the multiple questions and response options seen on paper. The evidence has consistently shown that PROMs are robust in regard to changes in mode of data collection, and even so called ‘significant’ changes in administration and visual layout have consistently shown to provide comparable data.

However, considering the importance of the data being captured, increasing the evidence base will facilitate the development of increasingly user-centric solutions through which there is potential to improve data quality. Without data we can trust, we cannot bring safe and effective treatments to *all* individuals. This will also be key to having regulatory agency confidence in ePROM implementations that adopt accessible practices that could be deemed a significant departure from the paper format.

It would be remiss to discuss ePROM implementation without reference to the authors and copyright holders of these measures, who often have their own requirements for implementation that are sometimes at odds with both ePROM best practices *and* accessibility best practices. This dynamic can present a challenge to improving the participant experience in clinical trials. For example, some require that the anchor text associated with the extremes of a horizontal NRS must not extend past the length of the highest and lowest numbers into the middle of the scale (i.e., the number 0 and number 10 on an 11-point scale); making the anchor text smaller, text-wrapping and/or splitting single words across lines to meet this requirement can run the risk of causing readability issues and fly in the face of accessibility guidelines (see Fig. 2 for examples).

Given the time-sensitive nature of the trial set-up process, discussions with measure authors/copyright holders around electronic implementations that deviate from ‘the norm’ but are arguably more accessible (whilst maintaining the integrity of a measure) often fall by the wayside; this **must** change for us to progress. As an industry, the goal of all stakeholders involved should be to make electronic data capture more accessible and usable, leading to more representative trials that are lower burden for patients. We should ask ourselves if we have lost sight of the intention of these measures? They are developed for use in specific therapeutic populations, yet instrument owner’s electronic implementation guidelines can sometimes make them difficult to use for those exact populations. Whilst this article focused on the electronic implementation as opposed to the instrument itself, it is clear that accessibility and universal design best practices start from the point of PROM development - whatever format that may be – to ensure a fit-for-purpose, valid, *and* accessible PROM. We must get to a point where accessibility best practices *are* (e)PROM best practices.

Finally, the criticality of consensus-based best practices was highlighted in Mowlem et al. (2024) [11], and it is important to acknowledge that the aim of this article is not to recreate and dictate new best practices, as the WCAG and 508 accessibility success criteria already exist. Rather, it was to identify where existing accessibility guidelines may not currently be being met in ePROM design and where they should be applied, and where further evidence is required before they are more-formally integrated into ePROM best practices. It is important that best practices are updated as the evidence-base advances. Due to the ongoing evolution of technologies used in clinical trials, and the related iterative nature of best practices developments, repeated peer-review publications of updated best practices may not be timely or practical. Instead, the authors suggest hosting of consensus developed best practice documents on the websites of relevant independent organizations, such as the Critical Path Institute’s (C-Path) Electronic Clinical Outcome Assessment Consortium (eCOAC), may be more appropriate, with peer-review publications limited to significant updates in best practices.

## Supplementary Information

Below is the link to the electronic supplementary material.

Supplementary Material 1

## Data Availability

Not applicable.
