# Supplementary material for: Accessible data collection methods in clinical trials: do current best practices for the implementation of electronic patient-reported outcome measures (ePROMs) meet accessibility standards?
Source: J Patient Rep Outcomes. 2026 Mar 25;10:63. doi: 10.1186/s41687-026-01039-8 (PMC13090402; doi:10.1186/s41687-026-01039-8)
Supplement: Supplementary file 1 — Supplementary Material 1 [file 41687_2026_1039_MOESM1_ESM.docx]

**Supplementary Material**

**Accessible data collection methods in clinical trials: Do current best practices for the implementation of electronic patient-reported outcome measures (ePROMs) meet accessibility standards?**

Supplementary Table 1. WCAG 2.2 accessibility success criteria as applied to screen-based ePROM implementations, including if they are relevant to migration practices, the system user interface, or core functionality of the system, if the given success criteria are currently addressed in ePROM best practices, and the associated considerations for ePROM best practices.

| **WCAG guideline & corresponding success criteria** | | **Category *(Migration, Core system functionality, UI)* *** | **Comment** | **Considerations for ePROM best practices** |
| --- | --- | --- | --- | --- |
| *1.1 Provide text alternatives for any non-text content so that it can be changed into other forms people need, such as large print, braille, speech, symbols or simpler language.* | |  |  |  |
| A | 1.1.1 Non-text Content  All [non-text content](https://www.w3.org/WAI/WCAG21/Understanding/non-text-content#dfn-non-text-content) that is presented to the user has a [text alternative](https://www.w3.org/WAI/WCAG21/Understanding/non-text-content#dfn-text-alternative) that serves the equivalent purpose, except for the situations listed below.  **Controls, Input**  If non-text content is a control or accepts user input, then it has a [name](https://www.w3.org/WAI/WCAG21/Understanding/non-text-content#dfn-name) that describes its purpose. (Refer to [Success Criterion 4.1.2](https://www.w3.org/WAI/WCAG21/Understanding/non-text-content#name-role-value) for additional requirements for controls and content that accepts user input.)  **Time-Based Media**  If non-text content is time-based media, then text alternatives at least provide descriptive identification of the non-text content. (Refer to [Guideline 1.2](https://www.w3.org/WAI/WCAG21/Understanding/non-text-content#time-based-media) for additional requirements for media.)  **Test**  If non-text content is a test or exercise that would be invalid if presented in [text](https://www.w3.org/WAI/WCAG21/Understanding/non-text-content#dfn-text), then text alternatives at least provide descriptive identification of the non-text content.  **Sensory**  If non-text content is primarily intended to create a [specific sensory experience](https://www.w3.org/WAI/WCAG21/Understanding/non-text-content#dfn-specific-sensory-experience), then text alternatives at least provide descriptive identification of the non-text content.  [**CAPTCHA**](https://www.w3.org/WAI/WCAG21/Understanding/non-text-content#dfn-captcha)  If the purpose of non-text content is to confirm that content is being accessed by a person rather than a computer, then text alternatives that identify and describe the purpose of the non-text content are provided, and alternative forms of CAPTCHA using output modes for different types of sensory perception are provided to accommodate different disabilities.  **Decoration, Formatting, Invisible**  If non-text content is [pure decoration](https://www.w3.org/WAI/WCAG21/Understanding/non-text-content#dfn-pure-decoration), is used only for visual formatting, or is not presented to users, then it is implemented in a way that it can be ignored by [assistive technology](https://www.w3.org/WAI/WCAG21/Understanding/non-text-content#dfn-assistive-technology). | Migration | This is not directly referred to in existing ePROM implementation best practices for screen-based devices, which focus on migration of the paper, typically text, format to electronic format.  Where non-text content is part of a PROM, such as images, this content is migrated *as is* to ensure ‘faithful migration’ and maintenance of the original format and its measurement properties. Unless the copyright holder has specified a text alternative for this content that has been validated, then those implementing electronically would not be able to program a validated text alternative.  A common response scale type, the Visual Analogue Scale (VAS), by its very definition (a line of fixed length, with text anchors at the extreme ends of the scale, but not words describing intermediate positions) would not allow for a text alternative. | For existing PROMs authors should create and validate text alternatives for non-text content that can then be provided when the measure is licensed for electronic implementation.  For new PROMs, create text alternatives for images at the time of measure creation and do not include visual analogue scales as a response type. |
| *1.2 Provide alternatives for time-based media.* | |  |  |  |
| A | [1.2.1 Audio-only and Video-only (Prerecorded)](https://www.w3.org/WAI/WCAG21/Understanding/audio-only-and-video-only-prerecorded) | N/A | This does not apply to ePROMs as they do not use pre-recorded audio or video content that would not be a media alternative for text. | N/A |
| A | 1[.2.2 Captions (Prerecorded)](https://www.w3.org/WAI/WCAG21/Understanding/captions-prerecorded) | N/A | This does not apply to ePROMs as they do not use pre-recorded video content. | N/A |
| A | [1.2.3 Audio Description or Media Alternative (Prerecorded)](https://www.w3.org/WAI/WCAG21/Understanding/audio-description-or-media-alternative-prerecorded) | N/A | This does not apply to ePROMs as they do not use pre-recorded video content. | N/A |
| AA | [1.2.4 Captions (Live)](https://www.w3.org/WAI/WCAG21/Understanding/captions-live) | N/A | This does not apply to ePROMs as live audio content in videos is not used. | N/A |
| AA | [1.2.5 Audio Description (Prerecorded)](https://www.w3.org/WAI/WCAG21/Understanding/audio-description-prerecorded) | N/A | This does not apply to ePROMs as they do not use pre-recorded video content. | N/A |
| AAA | [1.2.6 Sign Language (Prerecorded)](https://www.w3.org/WAI/WCAG21/Understanding/sign-language-prerecorded) | N/A | This does not apply to ePROMs as pre-recorded audio content in synchronized media is not used. | N/A |
| AAA | [1.2.7 Extended Audio Description (Prerecorded)](https://www.w3.org/WAI/WCAG21/Understanding/extended-audio-description-prerecorded) | N/A | This does not apply to ePROMs as pre-recorded audio content in synchronized media is not used. | N/A |
| AAA | [1.2.8 Media Alternative (Prerecorded)](https://www.w3.org/WAI/WCAG21/Understanding/media-alternative-prerecorded) | N/A | This does not apply to ePROMs as use pre-recorded synchronized medial or pre-recorded video content. | N/A |
| AAA | [1.2.9 Audio-only (Live)](https://www.w3.org/WAI/WCAG21/Understanding/audio-only-live) | N/A | This does not apply to ePROMs as they do not use live audio-only content. | N/A |
| *1.3 Create content that can be presented in different ways (for example simpler layout) without losing information or structure.* | |  |  |  |
| A | [1.3.1 Info and Relationships](https://www.w3.org/WAI/WCAG21/Understanding/info-and-relationships) Information, [structure](https://www.w3.org/WAI/WCAG21/Understanding/info-and-relationships#dfn-structure), and [relationships](https://www.w3.org/WAI/WCAG21/Understanding/info-and-relationships#dfn-relationships) conveyed through [presentation](https://www.w3.org/WAI/WCAG21/Understanding/info-and-relationships#dfn-presentation) can be [programmatically determined](https://www.w3.org/WAI/WCAG21/Understanding/info-and-relationships#dfn-programmatically-determined) or are available in text. | Migration, UI | This is not directly referred to in existing ePROM best practices but the core system functionality should support this. | The ePRO system should be able to support the programmatic determination of any information, structure, and relationships conveyed through visual presentation (e.g., user interface component labels are associated with the fields they are labeling, headings are tagged, lists can be identified with list markup). |
| A | [1.3.2 Meaningful Sequence](https://www.w3.org/WAI/WCAG21/Understanding/meaningful-sequence)  When the sequence in which content is presented affects its meaning, a [correct reading sequence](https://www.w3.org/WAI/WCAG21/Understanding/meaningful-sequence#dfn-correct-reading-sequence) can be [programmatically determined](https://www.w3.org/WAI/WCAG21/Understanding/meaningful-sequence#dfn-programmatically-determined). | Core system functionality | An important part of PROM development and design includes the order of PRO questions and the order of the elements within an item. Therefore, the sequence in which the information is presented for a given PROM and its items is meaningful.  Best practices to ensure a faithful migration involve maintaining this sequence, including response options being presented in the same order as the original format. Generally, a single item per screen is implemented as best practices to facilitate consistent visual presentation across modalities and device types. In some instances, multiple items per screen may be implemented, and this should follow the same meaningful sequence as on the paper format it is being migrated from. | The ePRO system should be able to support programming of the intended sequence of content when implementing an ePROM, to ensure the meaning of the content is maintained and the same for those using assistive technologies. |
| A | [1.3.3 Sensory Characteristics](https://www.w3.org/WAI/WCAG21/Understanding/sensory-characteristics)  Instructions provided for understanding and operating content do not rely solely on sensory characteristics of components such as shape, color, size, visual location, orientation, or sound. | Migration, UI | This is not directly referred to in ePROM best practices, but faithful migration involves no changes being made to the core wording of the measure (i.e., only implement minor changes to measure wording to align with the new mode of administration). If instructional text of the original measure refers to graphics, or uses only sensory characteristics for understanding content, then this would also be the case in the electronic implementation.  As detailed in 1.1.1, unless the copyright holder has specified a text alternative for this content that has been validated, then those implementing electronically would not be able to program a validated text alternative. | For authors of existing PROMs, if their measures include instructional text that relies solely on sensory characteristics, they should consider creating and validating versions of their measures that provide additional information to clarify instructions that are dependent on this kind of information.  For new PROMs, authors should ensure that they do not include content that relies solely on sensory characteristics.  With regard to the eCOA system UI, any reference to components (e.g., navigation buttons, menus), should ensure that users do not have to rely solely on sensory characteristics of the components to understand and operate them. This should also be considered in any training modules. |
| AA | [1.3.4 Orientation](https://www.w3.org/WAI/WCAG21/Understanding/orientation)  Content does not restrict its view and operation to a single display orientation, such as portrait or landscape, unless a specific display orientation is [essential](https://www.w3.org/WAI/WCAG21/Understanding/orientation#dfn-essential).  **Note**  Examples where a particular display orientation may be essential are a bank check, a piano application, slides for a projector or television, or virtual reality content where content is not necessarily restricted to landscape or portrait display orientation.  Mobile specific  Mobile application developers should try to support both orientations. If it is not possible to support both orientations, developers should ensure that it is easy for all users to change the orientation to return to a point at which their device orientation is supported.  Changes in orientation must be programmatically exposed to ensure detection by assistive technology such as screen readers. For example, if a screen reader user is unaware that the orientation has changed the user might perform incorrect navigation commands. | Migration, Core system functionality | ePROM best practice is to keep the orientation consistent, and oftentimes ‘locked’, with the default being portrait orientation for mobile devices. Further, copyright holder guidelines on e-implementation (if available) often specify the orientation must be 'locked' to the dictated orientation for the given electronic mode.  However, no evidence can be identified that claims content would only be understood in a certain orientation; whilst the aim of a consistently applied orientation is to facilitate standardization and reduce any potential frustration users may experience from orientation switching, it could lead to challenges for certain populations, such as those who have dexterity impairments and use a mounted device in a specific orientation. | Users should not be restricted to an enforced orientation and should be given the ability to switch to their preferred orientation. If this leads to the need for scrolling, then this should be implemented with the relevant functionality as outlined in Mowlem et al. (2024).^6^ Any changes in orientation must be programmatically exposed to ensure assistive technologies can detect it.  Further research may be required to mitigate concerns that this could impact the measurement properties of the PROM. |
| AA | [1.3.5 Identify Input Purpose](https://www.w3.org/WAI/WCAG21/Understanding/identify-input-purpose)  The purpose of each input field collecting information about the user can be [programmatically determined](https://www.w3.org/WAI/WCAG21/Understanding/identify-input-purpose#dfn-programmatically-determined) when:   - The input field serves a purpose identified in the [Input Purposes for user interface components section](https://www.w3.org/WAI/WCAG21/Understanding/identify-input-purpose#input-purposes); and - The content is implemented using technologies with support for identifying the expected meaning for form input data. | UI | ePROMs do not collect the content types covered by this success criteria; however, ePRO systems should be secure and require a password to login, an input type that is covered. | N/A |
| AAA | [1.3.6 Identify Purpose](https://www.w3.org/WAI/WCAG21/Understanding/identify-purpose)  In content implemented using markup languages, the purpose of [user interface components](https://www.w3.org/WAI/WCAG21/Understanding/identify-purpose#dfn-user-interface-component), icons, and [regions](https://www.w3.org/WAI/WCAG21/Understanding/identify-purpose#dfn-region) can be [programmatically determined](https://www.w3.org/WAI/WCAG21/Understanding/identify-purpose#dfn-programmatically-determined). | N/A | N/A | N/A |
| *1.4 Make it easier for users to see and hear content including separating foreground from background.* | |  |  |  |
| A | [1.4.1 Use of Color](https://www.w3.org/WAI/WCAG21/Understanding/use-of-color) Color is not used as the only visual means of conveying information, indicating an action, prompting a response, or distinguishing a visual element.  **Note**  This success criterion addresses color perception specifically. Other forms of perception are covered in [Guideline 1.3](https://www.w3.org/WAI/WCAG21/Understanding/use-of-color#adaptable) including programmatic access to color and other visual presentation coding. | Migration, UI | This is not directly referred to in existing ePROM implementation best practices, but best practices focus on faithful migration of the paper format to electronic format, and if PROMs have been developed where color alone is used to convey information, they would be migrated in this way to maintain the measurement properties of the PROM.  The eCOA provider has control over the colors used for the user interface, such as the navigation buttons. | For existing PROMs that include color as the only way to convey certain information, authors should create and validate versions where this is not the case.  For new PROMs, authors should not use only color as a means to convey information.  Within the UI, eCOA vendors should not only use color as a means to convey information. |
| A | [1.4.2 Audio Control](https://www.w3.org/WAI/WCAG21/Understanding/audio-control) | N/A | This does not apply to ePROMs, as measures do not include automatic playing of audio. If an interactive web or voice response system was being used as a mode of administration, it would not be used at the same time as a screen reader. | N/A |
| AA | [1.4.3 Contrast (Minimum)](https://www.w3.org/WAI/WCAG21/Understanding/contrast-minimum)  The visual presentation of [text](https://www.w3.org/WAI/WCAG21/Understanding/contrast-minimum#dfn-text) and [images of text](https://www.w3.org/WAI/WCAG21/Understanding/contrast-minimum#dfn-image-of-text) has a [contrast ratio](https://www.w3.org/WAI/WCAG21/Understanding/contrast-minimum#dfn-contrast-ratio) of at least 4.5:1, except for the following:  **Large Text**  [Large-scale](https://www.w3.org/WAI/WCAG21/Understanding/contrast-minimum#dfn-large-scale) text and images of large-scale text have a contrast ratio of at least 3:1;  **Incidental**  Text or images of text that are part of an inactive [user interface component](https://www.w3.org/WAI/WCAG21/Understanding/contrast-minimum#dfn-user-interface-component), that are [pure decoration](https://www.w3.org/WAI/WCAG21/Understanding/contrast-minimum#dfn-pure-decoration), that are not visible to anyone, or that are part of a picture that contains significant other visual content, have no contrast requirement.  **Logotypes**  Text that is part of a logo or brand name has no contrast requirement.  Mobile specific  Mobile device content is viewed on smaller screens and in different conditions so this allowance for lessened contrast on large text must be considered carefully for mobile apps. For instance, the default point size for mobile platforms might be larger than the default point size used on non-mobile devices. When determining which contrast ratio to follow, developers should strive to make sure to apply the lessened contrast ratio only when text is roughly equivalent to 1.2 times bold or 1.5 times (120% bold or 150%) that of the default platform size. Note, however, that the use of text that is 1.5 times the default on mobile platforms does not imply that the text will be readable by a person with low vision. People with low vision will likely need and use additional platform level accessibility features and assistive technology such as increased text size and zoom features to access mobile content. | UI | This is not directly referred to in existing ePROM implementation best practices and should be applied to the entire UI of the system. | The ePRO system should ensure a contrast ratio of at least 4.5:1 for text and images of text, and 3:1 for large-scale text and images of large-scale-text.  For mobile devices, the ePRO system should strive to have the contrast ratio set out in 1.4.6. |
| AA | [1.4.4 Resize Text](https://www.w3.org/WAI/WCAG21/Understanding/resize-text)  Except for [captions](https://www.w3.org/WAI/WCAG21/Understanding/resize-text#dfn-captions) and [images of text](https://www.w3.org/WAI/WCAG21/Understanding/resize-text#dfn-image-of-text), [text](https://www.w3.org/WAI/WCAG21/Understanding/resize-text#dfn-text) can be resized without [assistive technology](https://www.w3.org/WAI/WCAG21/Understanding/resize-text#dfn-assistive-technology) up to 200 percent without loss of content or functionality.  Mobile specific  The following methods might be used:   - Ensure that the browser pinch zoom is not blocked by the page's viewport meta element so that it can be used to zoom the page to 200%. Restrictive values for user-scalable and maximum-scale attributes of this meta element should be avoided. *Note:* Relying on full viewport zooming (e.g. not blocking the browser's pinch zoom feature) requires the user to pan horizontally as well as vertically. While this technique meets the success criteria it is less usable than supporting text resizing features that reflow content to the user's chosen viewport size. It is best practice to use techniques that support text resizing without requiring horizontal panning. - Support for system fonts that follow platform level user preferences for text size. - Provide on-page controls   A variety of methods allow the user to control content size on mobile devices with small screens.   - OS-level features   - Set default text size (typically controlled from the Display Settings) *Note*: System text size is often not supported by mobile browsers.   - Magnify entire screen (typically controlled from the Accessibility Settings). *Note*: Using this setting requires the user to pan vertically and horizontally.   - Magnifying lens view under user's finger (typically controlled from the Accessibility Settings) - Browser-level features   - Set default text size of text rendered in the browser's viewport     - Reading mode that renders main content at a user-specified text size   - Magnify browser's viewport (typically "pinch-zoom"). *Note*: Using this setting requires the user to pan vertically and horizontally.     - *Note*: Some browsers have features that might modify this type of magnification (e.g. re-flowing the content at the new magnification level, overriding author attempts to prevent pinch-zoom). | Migration, Core system functionality, UI | Current ePROM best practice is to keep font size consistent within a single PROM and between PROMs on the same device where possible, though acknowledge that limitations in the size of the device may make it necessary to adjust the font size between individual measures or even screens within a PROM. Further, there should be consideration around not making text so small it is unreadable, and in this instance enable scrolling; however, this may still not satisfy requirements for some users.  However, this is still under the control of the ePRO system, and established during the migration process, as opposed to allowing the user to alter the font size, which is generally not enabled. Some have even recommended disabling the ability for users to over-ride specific app settings, including font size^11^.  Regulatory agencies, such as FDA, state that administrator or respondent burden will be reviewed to ensure quality and completeness of COA data, which includes font size that is too small to read easily, and encourages consideration around the use of large text for certain populations.^26^  Smartphone devices tend to use pinch and zoom; however, this can lead to vertical and horizontal scrolling which may be problematic for usability and not recommended in ePROM best practices. | Given that scrolling is recommended over making content so small it creates readability issues, enabling 200% text resizing that does not lead to loss of functionality or content, along with the appropriate scrolling functionality for the reflow, is recommended.  It is recommended that research is conducted to understand the most optimal way to increase text size, including zooming and its impact on the measurement properties of a PROM, especially given not everyone will use assistive technologies. Early work on this topic has shown measurement comparability when allowing users to zoom ^26^. |
| AA | [1.4.5 Images of Text](https://www.w3.org/WAI/WCAG21/Understanding/images-of-text) | N/A | This does not apply to ePROMs as images of text are not used in the absence of other significant visual content. | N/A |
| AAA | [1.4.6 Contrast (Enhanced)](https://www.w3.org/WAI/WCAG21/Understanding/contrast-enhanced)  The visual presentation of [text](https://www.w3.org/WAI/WCAG21/Understanding/contrast-enhanced#dfn-text) and [images of text](https://www.w3.org/WAI/WCAG21/Understanding/contrast-enhanced#dfn-image-of-text) has a [contrast ratio](https://www.w3.org/WAI/WCAG21/Understanding/contrast-enhanced#dfn-contrast-ratio) of at least 7:1, except for the following:  **Large Text**  [Large-scale](https://www.w3.org/WAI/WCAG21/Understanding/contrast-enhanced#dfn-large-scale) text and images of large-scale text have a contrast ratio of at least 4.5:1;  **Incidental**  Text or images of text that are part of an inactive [user interface component](https://www.w3.org/WAI/WCAG21/Understanding/contrast-enhanced#dfn-user-interface-component), that are [pure decoration](https://www.w3.org/WAI/WCAG21/Understanding/contrast-enhanced#dfn-pure-decoration), that are not visible to anyone, or that are part of a picture that contains significant other visual content, have no contrast requirement.  **Logotypes**  Text that is part of a logo or brand name has no contrast requirement. | UI | As detailed in 1.4.3, this is not directly referred to in existing ePROM implementation best practices. Further, ePROMs do not use a large amount of variation within a single screen, and so this criteria is likely not deemed necessary (especially given in is AAA). | If possible, the ePRO system should meet this contrast criteria – especially for mobile devices - but 1.4.3 is sufficient. |
| AAA | [1.4.7 Low or No Background Audio](https://www.w3.org/WAI/WCAG21/Understanding/low-or-no-background-audio) | N/A | This does not apply to ePROMs, as they do not use background audio. | N/A |
| AAA | [1.4.8 Visual Presentation](https://www.w3.org/WAI/WCAG21/Understanding/visual-presentation)  For the visual presentation of [blocks of text](https://www.w3.org/WAI/WCAG21/Understanding/visual-presentation#dfn-blocks-of-text), a [mechanism](https://www.w3.org/WAI/WCAG21/Understanding/visual-presentation#dfn-mechanism) is available to achieve the following:   - Foreground and background colors can be selected by the user. - Width is no more than 80 characters or glyphs (40 if CJK). - Text is not justified (aligned to both the left and the right margins). - Line spacing (leading) is at least space-and-a-half within paragraphs, and paragraph spacing is at least 1.5 times larger than the line spacing. - Text can be resized without assistive technology up to 200 percent in a way that does not require the user to scroll horizontally to read a line of text [on a full-screen window](https://www.w3.org/WAI/WCAG21/Understanding/visual-presentation#dfn-on-a-full-screen-window). | Migration, Core system functionality, UI | The ability for the foreground and background colour to be selected by the user is not something currently called out in ePROM best practices, and is considered part of the UI and not the PROM itself.  Text alignment is not directly referred to in existing implementation best practices but migrations usually involve maintenance of the text alignment with the original format and copyright holder/license holder electronic implementation guidance often dictate this. Number of characters per line is also not detailed in best practices.  There is not currently a specified ePROM implementation best practice for line spacing and it is often left to the vendor discretion or the copyright holder/license holder electronic implementation guidance may dictate this.  Furthermore, line spacing, along with text/font size will be reduced if scrolling is not allowed. Current ePROM implementation best practice is to enable scrolling rather than reducing the font size to avoid causing reading difficulties.  Resizing of text on a full-screen window refers to the most common sized desktop/laptop display with the viewport maximised and so this is not a requirement for mobile devices, which would use pinch and zoom. | Provide mechanisms to allow the user to:   - select the foreground and background colours, to aid comprehension for those with certain visual or cognitive disabilities (this goes beyond dark mode). - enabling 200% text resizing for larger devices that does not lead to loss of functionality or content if zoom is not available; smartphone devices tend to use pinch and zoom (see 1.4.4)   Do not implement PROMs using justified text alignment – conversation with copyright holders may be required if they dictate this, and where possible ensure the width of blocks of text is no more than 80 characters or glyphs.  Where possible, have standardized line spacing in all ePROs of at least space-and-a-half within paragraphs, and paragraph spacing is at least 1.5 times larger than the line spacing. |
| AAA | [1.4.9 Images of Text (No Exception)](https://www.w3.org/WAI/WCAG21/Understanding/images-of-text-no-exception) | N/A | As above (1.4.5) | N/A |
| AA | [1.4.10 Reflow](https://www.w3.org/WAI/WCAG21/Understanding/reflow)  Content can be presented without loss of information or functionality, and without requiring scrolling in two dimensions for:   - Vertical scrolling content at a width equivalent to 320 [CSS pixels](https://www.w3.org/WAI/WCAG21/Understanding/reflow#dfn-css-pixel); - Horizontal scrolling content at a height equivalent to 256 [CSS pixels](https://www.w3.org/WAI/WCAG21/Understanding/reflow#dfn-css-pixel).   Except for parts of the content which require two-dimensional layout for usage or meaning.  **Note**  320 CSS pixels is equivalent to a starting viewport width of 1280 CSS pixels wide at 400% zoom. For web content which is designed to scroll horizontally (e.g., with vertical text), 256 CSS pixels is equivalent to a starting viewport height of 1024 CSS pixels at 400% zoom.  **Note**  Examples of content which requires two-dimensional layout are images required for understanding (such as maps and diagrams), video, games, presentations, data tables (not individual cells), and interfaces where it is necessary to keep toolbars in view while manipulating content. It is acceptable to provide two-dimensional scrolling for such parts of the content.  Mobile specific  The small screen size on many mobile devices limits the amount of content that can be displayed without scrolling.  Positioning important page information so it is visible without requiring scrolling can assist users with low vision and users with cognitive impairments.  If a user with low vision has the screen magnified only a small portion of the page might be viewable at a given time. Placing important elements before the page scroll allows those who use screen magnifiers to locate important information without having to scroll the view to move the magnified area. Placing important elements before the page scroll also makes it possible to locate content without performing an interaction. This assists users that have cognitive impairments such as short-term memory disabilities | Migration, Core system functionality | Current ePROM best practice is to make items self-contained when they are being implemented as a single item per screen, and to enable vertical scrolling as opposed to reducing font to a size that would cause reading difficulties, with the relevant scrolling functionality. Two-dimensional scrolling is not a recommended best practice.  However, many copyright/license holders still do not allow vertical scrolling when implementing their measures electronically, and some sponsors also request no scrolling enabled.  Placing important information before the page scroll may not be possible with ePROM, as items should be presented in the order of the original format. | Maintain existing best practices. |
| AA | [1.4.11 Non-text Contrast](https://www.w3.org/WAI/WCAG21/Understanding/non-text-contrast)  The visual [presentation](https://www.w3.org/WAI/WCAG21/Understanding/non-text-contrast#dfn-presentation) of the following have a [contrast ratio](https://www.w3.org/WAI/WCAG21/Understanding/non-text-contrast#dfn-contrast-ratio) of at least 3:1 against adjacent color(s):  **User Interface Components**  Visual information required to identify [user interface components](https://www.w3.org/WAI/WCAG21/Understanding/non-text-contrast#dfn-user-interface-component) and [states](https://www.w3.org/WAI/WCAG21/Understanding/non-text-contrast#dfn-state), except for inactive components or where the appearance of the component is determined by the user agent and not modified by the author;  **Graphical Objects**  Parts of graphics required to understand the content, except when a particular presentation of graphics is [essential](https://www.w3.org/WAI/WCAG21/Understanding/non-text-contrast#dfn-essential) to the information being conveyed. | Migration, UI | The contrast ratio used to identify user interface components and their states and graphics (e.g., measures that include body maps or faces), is not something that is documented in ePROM best practices. The identification of user interface components (e.g., control buttons) is part of the UI of the eCOA provider. The indication of state would not only apply to when a control has been selected, but also when a response option has been selected by the user. | Where ePROMs include graphics, and in instances where visual information is the only means to identify a control, or where visual information is used to indicate state, ensure a contrast ratio of 3:1 for the colour of user interface components against adjacent colours. |
| AA | [1.4.12 Text Spacing](https://www.w3.org/WAI/WCAG21/Understanding/text-spacing)  In content implemented using markup languages that support the following [text](https://www.w3.org/WAI/WCAG21/Understanding/text-spacing#dfn-text) [style properties](https://www.w3.org/WAI/WCAG21/Understanding/text-spacing#dfn-style-property), no loss of content or functionality occurs by setting all of the following and by changing no other style property:   - Line height (line spacing) to at least 1.5 times the font size; - Spacing following paragraphs to at least 2 times the font size; - Letter spacing (tracking) to at least 0.12 times the font size; - Word spacing to at least 0.16 times the font size.   Exception: Human languages and scripts that do not make use of one or more of these text style properties in written text can conform using only the properties that exist for that combination of language and script. | Migration, UI | This is not directly referred to in existing ePROM implementation best practices and standards should be applied to the entire UI of the system.  Some copyright holders/authors dictate certain spacing requirements or specify that the spacing of the electronic implementation must be matched with the paper format. However, this criterion is not about presenting it this way but ensuring that is the user is able to set the text spacing to those outlined, there is no loss of content or functionality. Previously, it has been suggested that eCOA systems should be set up to over-ride the ability for users to make changes ^11,15^. | If the user is able set the text spacing to those outlined ensure this is without loss of content or functionality. |
| AA | [1.4.13 Content on Hover or Focus](https://www.w3.org/WAI/WCAG21/Understanding/content-on-hover-or-focus) | N/A | This does not apply to ePROMs as they do not present additional content if a component receives focus. | N/A |
| *2.1 Make all functionality available from a keyboard.* | |  |  |  |
| A | [2.1.1 Keyboard](https://www.w3.org/WAI/WCAG21/Understanding/keyboard)  All [functionality](https://www.w3.org/WAI/WCAG21/Understanding/keyboard#dfn-functionality) of the content is operable through a [keyboard interface](https://www.w3.org/WAI/WCAG21/Understanding/keyboard#dfn-keyboard-interface) without requiring specific timings for individual keystrokes, except where the underlying function requires input that depends on the path of the user's movement and not just the endpoints.  **Note**  This exception relates to the underlying function, not the input technique. For example, if using handwriting to enter text, the input technique (handwriting) requires path-dependent input but the underlying function (text input) does not.  **Note**  This does not forbid and should not discourage providing mouse input or other input methods in addition to keyboard operation. | UI | This is not detailed in ePROM best practices.  Specific elements of ePROMs (e.g., response scale types) do not depend on the path of the user’s movement. | eCOA systems should allow for all functionality of content to be operable though a keyboard interface. |
| A | [2.1.2 No Keyboard Trap](https://www.w3.org/WAI/WCAG21/Understanding/no-keyboard-trap)  If keyboard focus can be moved to a component of the page using a [keyboard interface](https://www.w3.org/WAI/WCAG21/Understanding/no-keyboard-trap#dfn-keyboard-interface), then focus can be moved away from that component using only a keyboard interface, and, if it requires more than unmodified arrow or tab keys or other standard exit methods, the user is advised of the method for moving focus away.  **Note**  Since any content that does not meet this success criterion can interfere with a user's ability to use the whole page, all content on the Web page (whether it is used to meet other success criteria or not) must meet this success criterion. See [Conformance Requirement 5: Non-Interference](https://www.w3.org/WAI/WCAG21/Understanding/no-keyboard-trap#cc5). | UI | This is not detailed in ePROM best practices. | If 2.1.1 is applicable, then this must be possible. That is, eCOA systems should allow for all functionality of content to be operable though a keyboard interface, and should ensure there is no “keyboard trap” that prevents focus being moved away from the component using the keyboard. |
| AAA | [2.1.3 Keyboard (No Exception)](https://www.w3.org/WAI/WCAG21/Understanding/keyboard-no-exception)  All [functionality](https://www.w3.org/WAI/WCAG21/Understanding/keyboard-no-exception#dfn-functionality) of the content is operable through a [keyboard interface](https://www.w3.org/WAI/WCAG21/Understanding/keyboard-no-exception#dfn-keyboard-interface) without requiring specific timings for individual keystrokes. | UI | This is not detailed in ePROM best practices. Given that the exception to 2.1.1 is not relevant to ePROM (that content does not use path-dependent input), this criterion would be met if 2.1.1 is. | See 2.1.1 |
| A | [2.1.4 Character Key Shortcuts](https://www.w3.org/WAI/WCAG21/Understanding/character-key-shortcuts)  If a [keyboard shortcut](https://www.w3.org/WAI/WCAG21/Understanding/character-key-shortcuts#dfn-keyboard-shortcut) is implemented in content using only letter (including upper- and lower-case letters), punctuation, number, or symbol characters, then at least one of the following is true:  **Turn off**  A [mechanism](https://www.w3.org/WAI/WCAG21/Understanding/character-key-shortcuts#dfn-mechanism) is available to turn the shortcut off;  **Remap**  A mechanism is available to remap the shortcut to include one or more non-printable keyboard keys (e.g., Ctrl, Alt);  **Active only on focus**  The keyboard shortcut for a [user interface component](https://www.w3.org/WAI/WCAG21/Understanding/character-key-shortcuts#dfn-user-interface-component) is only active when that component has focus. | UI | This does not apply to ePROMs, as they do not use character key shortcuts. | N/A |
| *2.2 Provide users enough time to read and use content.* | |  |  |  |
| A | [2.2.1 Timing Adjustable](https://www.w3.org/WAI/WCAG21/Understanding/timing-adjustable)  For each time limit that is set by the content, at least one of the following is true:  **Turn off**  The user is allowed to turn off the time limit before encountering it; or  **Adjust**  The user is allowed to adjust the time limit before encountering it over a wide range that is at least ten times the length of the default setting; or  **Extend**  The user is warned before time expires and given at least 20 seconds to extend the time limit with a simple action (for example, "press the space bar"), and the user is allowed to extend the time limit at least ten times; or  **Real-time Exception**  The time limit is a required part of a real-time event (for example, an auction), and no alternative to the time limit is possible; or  **Essential Exception**  The time limit is [essential](https://www.w3.org/WAI/WCAG21/Understanding/timing-adjustable#dfn-essential) and extending it would invalidate the activity; or  **20 Hour Exception**  The time limit is longer than 20 hours.  **Note**  This success criterion helps ensure that users can complete tasks without unexpected changes in content or context that are a result of a time limit. This success criterion should be considered in conjunction with [Success Criterion 3.2.1](https://www.w3.org/WAI/WCAG21/Understanding/timing-adjustable#on-focus), which puts limits on changes of content or context as a result of user action. | Core system functionality, UI | This is not detailed in ePROM best practices, but the time limit to complete a given ePROM is an important consideration requiring careful thought.  A benefit of electronic implementation is the contemporaneousness of the data – having a time and date stamp associated with it, and the time window for completion of an ePROM is an essential part of the event, it should not be unlimited, and in many cases can require completion in a time window far less than 20 hours.  PROMs are typically time-bound – patients are rating themselves over a specified period of time, or within the current moment, and the time window for completion of an ePROM is an essential part of the event. Within the context of a clinical trial, this time window should not be unlimited, and in many cases study design requires completion in a time window far less than 20 hours.  Currently, there is no agreed upon best practice for the length of time a user is inactive before logout or how much time they are allowed to take to complete a given ePROM within the defined time window. | Given the importance of the contemporaneousness of ePROM data, time limits should not be turned off. Instead, it is recommended that the ePRO system informs/warns users if they are about to be logged out due to inactivity, and given 20 seconds to extend the time limit with an action (such as “tap the screen”), and that the user is allowed to extend the time limit at least 10 times, given that it remains within the allocated time window as defined by the protocol.  Information on time limits for task completion and system logout due to inactivity should be included in participant training material. |
| A | [2.2.2 Pause, Stop, Hide](https://www.w3.org/WAI/WCAG21/Understanding/pause-stop-hide) | N/A | This does not apply to ePROMs, as they do not include moving, blinking, scrolling, or auto-updating that starts automatically. | N/A |
| AAA | [2.2.3 No Timing](https://www.w3.org/WAI/WCAG21/Understanding/no-timing)  Timing is not an [essential](https://www.w3.org/WAI/WCAG21/Understanding/no-timing#dfn-essential) part of the event or activity presented by the content, except for non-interactive [synchronized media](https://www.w3.org/WAI/WCAG21/Understanding/no-timing#dfn-synchronized-media) and [real-time events](https://www.w3.org/WAI/WCAG21/Understanding/no-timing#dfn-real-time-event). | N/A | This does not apply to ePROMs as timing is an essential component of their completion. | See 2.2.1 |
| AAA | [2.2.4 Interruptions](https://www.w3.org/WAI/WCAG21/Understanding/interruptions)  Interruptions can be postponed or suppressed by the user, except interruptions involving an [emergency](https://www.w3.org/WAI/WCAG21/Understanding/interruptions#dfn-emergency). | Core system functionality | This is not included in ePROM best practices. However, it is a concern for some sponsors with BYOD, as the user could be distracted by other notifications on their phone when completing an ePROM, though this criterion is detailed as being under the users control as opposed to the author of the solution. | Enable users to postpone any other notifications on their device when using the ePRO system, apart from alerts concerning emergencies. |
| AAA | [2.2.5 Re-authenticating](https://www.w3.org/WAI/WCAG21/Understanding/re-authenticating)  When an authenticated session expires, the user can continue the activity without loss of data after re-authenticating. | N/A | This is not included in ePROM best practices, but a benefit of electronic implementation is the contemporaneousness of the data – having a time and date stamp associated with it. Currently, most ePRO providers are set up so that if a user was to be logged out, the data would not be saved unless it had been submitted on completion of the measure, as unless they logged back on instantly, multiple time (and potentially dates) would be associated with the data and lead to uninterpretable data. As detailed in 2.2.1, users should be informed if a session is close to time out. | N/A |
| AAA | [2.2.6 Timeouts](https://www.w3.org/WAI/WCAG21/Understanding/timeouts)  Users are warned of the duration of any [user inactivity](https://www.w3.org/WAI/WCAG21/Understanding/timeouts#dfn-user-inactivity) that could cause data loss, unless the data is preserved for more than 20 hours when the user does not take any actions.  **Note**  Privacy regulations may require explicit user consent before user identification has been authenticated and before user data is preserved. In cases where the user is a minor, explicit consent may not be solicited in most jurisdictions, countries or regions. Consultation with privacy professionals and legal counsel is advised when considering data preservation as an approach to satisfy this success criterion. | Core system functionality | This is not detailed in ePROM best practices. In some systems, if a user exits an ePROM or logs out of the system prior to completion (i.e., they have only partially completed a measure and not submitted the data), the data points that were completed would not be saved and the user would not be able to pick-up where they left off once they log back. Whereas, in other systems, given they are still within the time window for completion of the measure as set out in the protocol and specified for the electronic build, the data entered thus far would be saved and they could pick up where they left off.  This is something that requires further thought and discussion, as the user could be in a different ‘state’, leading to unreliable and conflicting data between the different time points at which the different parts of a measure were completed^23^. Further, multiple time stamps (and potentially dates) could be associated with the data which could cause analysis problems.  With regard to accessibility, the important part for this criterion is that the user is warned of duration of any user inactivity that would lead to loss of the data they have entered so far. | Warn users of the duration of any user inactivity that would lead to loss of the data they have entered so far.  As outlined in 2.2.1, this should be included in any participant training material.  It is recommended that research is conducted on the impact of saving and completing later to the reliability of the data, and how much time sould be allowed should this be a chosen option. |
| *2.3 Do not design content in a way that is known to cause seizures* | |  |  |  |
| A | [2.3.1 Three Flashes or Below Threshold](https://www.w3.org/WAI/WCAG21/Understanding/three-flashes-or-below-threshold) | N/A | This does not apply to ePROMs. | N/A |
| AAA | [2.3.2 Three Flashes](https://www.w3.org/WAI/WCAG21/Understanding/three-flashes)  [Web pages](https://www.w3.org/WAI/WCAG21/Understanding/three-flashes#dfn-web-page) do not contain anything that [flashes](https://www.w3.org/WAI/WCAG21/Understanding/three-flashes#dfn-flash) more than three times in any one second period. | N/A | This does not apply to ePROMs. | N/A |
| AAA | [2.3.3 Animation from Interactions](https://www.w3.org/WAI/WCAG21/Understanding/animation-from-interactions) | N/A | This does not apply to ePROMs. | N/A |
| *2.4 Provide ways to help users navigate, find content, and determine where they are.* | |  |  |  |
| A | [2.4.1 Bypass Blocks](https://www.w3.org/WAI/WCAG21/Understanding/bypass-blocks) | N/A | This does not apply to ePROMs, as they do not contain repeated blocks of content across multiple screens. | N/A |
| A | [2.4.2 Page Titled](https://www.w3.org/WAI/WCAG21/Understanding/page-titled)  [Web pages](https://www.w3.org/WAI/WCAG21/Understanding/page-titled#dfn-web-page) have titles that describe topic or purpose. | UI | This is not detailed in ePROM best practices, and it not relevant to mobile apps, but would be relevant to web-based solutions. | Ensure the ePROM ‘page’ has a meaningful title specified in the TITLE element for web implementations. |
| A | [2.4.3 Focus Order](https://www.w3.org/WAI/WCAG21/Understanding/focus-order)  If a [Web page](https://www.w3.org/WAI/WCAG21/Understanding/focus-order#dfn-web-page) can be [navigated sequentially](https://www.w3.org/WAI/WCAG21/Understanding/focus-order#dfn-navigated-sequentially) and the navigation sequences affect meaning or operation, focusable components receive focus in an order that preserves meaning and operability. | Core system functionality | An important part of PROM development and design includes the order of PRO questions and the order of the elements within an item. Therefore, the sequence in which the information is presented for a given PROM and its items is meaningful.  Best practices to ensure a faithful migration involve maintaining this sequence, including response options being presented in the same order as the original format. Generally, a single item per screen is implemented as best practices to facilitate consistent visual presentation across modalities and device types. In some instances, multiple items per screen may be implemented, and this should follow the same meaningful sequence as on the paper format it is being migrated from. | The ePRO system should be programed to support logical movement (i.e., the intended meaningful sequence of content)  through the focusable components using only the keyboard, to ensure the meaning of the content is maintained. |
| A | [2.4.4 Link Purpose (In Context)](https://www.w3.org/WAI/WCAG21/Understanding/link-purpose-in-context)  The [purpose of each link](https://www.w3.org/WAI/WCAG21/Understanding/link-purpose-in-context#dfn-link-purpose) can be determined from the link text alone or from the link text together with its [programmatically determined link context](https://www.w3.org/WAI/WCAG21/Understanding/link-purpose-in-context#dfn-programmatically-determined-link-context), except where the purpose of the link would be [ambiguous to users in general](https://www.w3.org/WAI/WCAG21/Understanding/link-purpose-in-context#dfn-ambiguous-to-users-in-general). | N/A | This does not apply to ePROMs, as they do not contain links. | N/A |
| AA | [2.4.5 Multiple Ways](https://www.w3.org/WAI/WCAG21/Understanding/multiple-ways)  More than one way is available to locate a [Web page](https://www.w3.org/WAI/WCAG21/Understanding/multiple-ways#dfn-web-page) within a [set of Web pages](https://www.w3.org/WAI/WCAG21/Understanding/multiple-ways#dfn-set-of-web-pages) except where the Web Page is the result of, or a step in, a [process](https://www.w3.org/WAI/WCAG21/Understanding/multiple-ways#dfn-process). | UI | This does not apply, as items within an ePROM should be completed within a specified order. It would be applicable to the ePRO system design as a whole, as this often contains a menu with other items, such as training, site contact details. | N/A |
| AA | [2.4.6 Headings and Labels](https://www.w3.org/WAI/WCAG21/Understanding/headings-and-labels)  Headings and [labels](https://www.w3.org/WAI/WCAG21/Understanding/headings-and-labels#dfn-label) describe topic or purpose. | Migration | This does not apply exclusively to ePROMs but is relevant to the actual development of a COA which should be ‘fit-for-purpose' in the target population. PROMS do not always have a title or heading that is meaningful to users.  Response options would equate to labels in the electronic format, as they should describe the purpose of the associated field to the user, and again should have been developed to be ‘fit-for-purpose'. | N/A |
| AA | [2.4.7 Focus Visible](https://www.w3.org/WAI/WCAG21/Understanding/focus-visible)  Any keyboard operable user interface has a mode of operation where the keyboard focus indicator is visible. | UI | This is not called out in ePROM best practices and applies to the UI of the system as a whole. | When an ePRO system is keyboard operable, there should be a mode of operation where the keyboard focus indicator is visible. |
| AAA | [2.4.8 Location](https://www.w3.org/WAI/WCAG21/Understanding/location) | N/A | This does not apply to ePROMs. | N/A |
| AAA | [2.4.9 Link Purpose (Link Only)](https://www.w3.org/WAI/WCAG21/Understanding/link-purpose-link-only)  A [mechanism](https://www.w3.org/WAI/WCAG21/Understanding/link-purpose-link-only#dfn-mechanism) is available to allow the purpose of each link to be identified from link text alone, except where the purpose of the link would be [ambiguous to users in general](https://www.w3.org/WAI/WCAG21/Understanding/link-purpose-link-only#dfn-ambiguous-to-users-in-general). | N/A | This does not apply to ePROMs, as they do not contain links. | N/A |
| AAA | [2.4.10 Section Headings](https://www.w3.org/WAI/WCAG21/Understanding/section-headings)  [Section](https://www.w3.org/WAI/WCAG21/Understanding/section-headings#dfn-section) headings are used to organize the content.  **Note**  "Heading" is used in its general sense and includes titles and other ways to add a heading to different types of content.  **Note**  This success criterion covers sections within writing, not [user interface components](https://www.w3.org/WAI/WCAG21/Understanding/section-headings#dfn-user-interface-component). User interface components are covered under [Success Criterion 4.1.2](https://www.w3.org/WAI/WCAG21/Understanding/section-headings#name-role-value). | N/A | This does not apply to ePROMs but is relevant to the actual development of a measure; if section headings were included in the measure, then these would be migrated. | N/A |
| AA | [2.4.11 Focus Not Obscured (Minimum)](https://www.w3.org/WAI/WCAG22/Understanding/focus-not-obscured-minimum)  When a [user interface component](https://www.w3.org/WAI/WCAG22/Understanding/focus-not-obscured-minimum#dfn-user-interface-component) receives keyboard focus, the component is not entirely hidden due to author-created content.  **Note**  Where content in a configurable interface can be repositioned by the user, then only the initial positions of user-movable content are considered for testing and conformance of this Success Criterion.  **Note**  Content opened by the *user* may obscure the component receiving focus. If the user can reveal the focused component without advancing the keyboard focus, the component with focus is not considered visually hidden due to author-created content. | Core System Functionality, UI | This is not included in ePROM best practices, but if certain components are part of a measure, for example, if a date/time selection or dropdown menu was implemented, then when a user focuses on that component the content may go over the top of other content and obscure it. As they do not persist after being interacted with or once they are no longer the primary point of user interaction, they do not fail this criterion as they are not obscuring the new content with focus.  Any modal dialog boxes that are implemented (such as an edit check or pop-up) would not be applicable as even when they appear directly on top of an item with focus, the dialog takes the focus and thus the new item with focus is visible and prevents interaction outside the modal until it is dismissed. | Ensure components that obscure other content do not persist after being interacted with or once they are no longer the primary point of user interaction, so they do not obscure other content that then receives focus. |
| AAA | [2.4.12 Focus Not Obscured (Enhanced)](https://www.w3.org/WAI/WCAG22/Understanding/focus-not-obscured-enhanced)  When a [user interface component](https://www.w3.org/WAI/WCAG22/Understanding/focus-not-obscured-enhanced#dfn-user-interface-component) receives keyboard focus, no part of the component is hidden by author-created content. | Core System Functionality, UI | See 2.4.11 | See 2.4.11 |
| AAA | [2.4.13 Focus Appearance](https://www.w3.org/WAI/WCAG22/Understanding/focus-appearance)  When the keyboard [focus indicator](https://www.w3.org/WAI/WCAG22/Understanding/focus-appearance#dfn-focus-indicator) is visible, an area of the focus indicator meets all the following:   - is at least as large as the area of a 2 [CSS pixel](https://www.w3.org/WAI/WCAG22/Understanding/focus-appearance#dfn-css-pixel) thick [perimeter](https://www.w3.org/WAI/WCAG22/Understanding/focus-appearance#dfn-perimeter) of the unfocused component or sub-component, and - has a contrast ratio of at least 3:1 between the same pixels in the focused and unfocused states.   Exceptions:   - The focus indicator is determined by the [user agent](https://www.w3.org/WAI/WCAG22/Understanding/focus-appearance#dfn-user-agent) and cannot be adjusted by the author, or - The focus indicator and the indicator's background color are not modified by the author.   **Note**  What is perceived as the user interface component or sub-component (to determine the perimeter) depends on its visual [presentation](https://www.w3.org/WAI/WCAG22/Understanding/focus-appearance#dfn-presentation). The visual presentation includes the component's visible [content](https://www.w3.org/WAI/WCAG22/Understanding/focus-appearance#dfn-content), border, and component-specific background. It does not include shadow and glow effects outside the component's content, background, or border.  **Note**  Examples of sub-components that may receive a focus indicator are menu items in an opened drop-down menu, or focusable cells in a grid.  **Note**  Contrast calculations can be based on colors defined within the [technology](https://www.w3.org/WAI/WCAG22/Understanding/focus-appearance#dfn-technology) (such as HTML, CSS and SVG). Pixels modified by user agent resolution enhancements and anti-aliasing can be ignored. | UI | This is not called out in ePROM best practices and applies to the UI of the system as a whole. | In addition to 2.4.7 - when an ePRO system is keyboard operable, there should be a mode of operation where the keyboard focus indicator is visible – ensure the focus indicator is clearly visible and discernable. |
| *2.5 Make it easier for users to operate functionality through various inputs beyond keyboard.* | |  |  |  |
| A | [2.5.1 Pointer Gestures](https://www.w3.org/WAI/WCAG21/Understanding/pointer-gestures) All [functionality](https://www.w3.org/WAI/WCAG21/Understanding/pointer-gestures#dfn-functionality) that uses multipoint or path-based gestures for operation can be operated with a [single pointer](https://www.w3.org/WAI/WCAG21/Understanding/pointer-gestures#dfn-single-pointer) without a path-based gesture, unless a multipoint or path-based gesture is [essential](https://www.w3.org/WAI/WCAG21/Understanding/pointer-gestures#dfn-essential).  **Note**  This requirement applies to web content that interprets pointer actions (i.e. this does not apply to actions that are required to operate the user agent or assistive technology) | N/A | This does not apply to ePROMs, as they include response scale types where only the endpoints matter. | N/A |
| A | [2.5.2 Pointer Cancellation](https://www.w3.org/WAI/WCAG21/Understanding/pointer-cancellation)  For [functionality](https://www.w3.org/WAI/WCAG21/Understanding/pointer-cancellation#dfn-functionality) that can be operated using a [single pointer](https://www.w3.org/WAI/WCAG21/Understanding/pointer-cancellation#dfn-single-pointer), at least one of the following is true:  **No Down-Event**  The [down-event](https://www.w3.org/WAI/WCAG21/Understanding/pointer-cancellation#dfn-down-event) of the pointer is not used to execute any part of the function;  **Abort or Undo**  Completion of the function is on the [up-event](https://www.w3.org/WAI/WCAG21/Understanding/pointer-cancellation#dfn-up-event), and a [mechanism](https://www.w3.org/WAI/WCAG21/Understanding/pointer-cancellation#dfn-mechanism) is available to abort the function before completion or to undo the function after completion;  **Up Reversal**  The up-event reverses any outcome of the preceding down-event;  **Essential**  Completing the function on the down-event is [essential](https://www.w3.org/WAI/WCAG21/Understanding/pointer-cancellation#dfn-essential).  **Note**  Functions that emulate a keyboard or numeric keypad key press are considered essential.  **Note**  This requirement applies to web content that interprets pointer actions (i.e. this does not apply to actions that are required to operate the user agent or assistive technology).  Mobile specific  Activating elements via the mouseup or touchend event. Using the mouseup or touchend event to trigger actions helps prevent unintentional actions during touch and mouse interaction. Mouse users clicking on actionable elements (links, buttons, submit inputs) should have the opportunity to move the cursor outside the element to prevent the event from being triggered. This allows users to change their minds without being forced to commit to an action. In the same way, elements accessed via touch interaction should generally trigger an event (e.g. navigation, submits) only when the touchend event is fired (i.e. when all of the following are true: the user has lifted the finger off the screen, the last position of the finger is inside the actionable element, and the last position of the finger equals the position at touchstart). | UI, Core System Functionality | This is not called out in ePROM best practices and applies to the UI of the system as a whole.   It is also relevant to the core system functionality to support the implementation of ePROMs, as how a response option is selected and aborted or undone should be considered. In implementations where users are allowed to skip an item, it is important to consider when a user has selected a response option and then wants to “unselect” it so that they can skip the item. For response scale types such as the VRS and NRS, clicking/taping on the response option already selected could deselect it; however, for response scales types such as the VAS or a numeric entry, this method would not be likely to work for undoing their selection. A possible option would be to have a ‘reset screen’ button for users to return the item to its original presentation (i.e., with no response selected). | Ensure that all response scale types within an ePROM have a way for selection to be aborted or cancelled by the user. |
| A | [2.5.3 Label in Name](https://www.w3.org/WAI/WCAG21/Understanding/label-in-name)  For [user interface components](https://www.w3.org/WAI/WCAG21/Understanding/label-in-name#dfn-user-interface-component) with [labels](https://www.w3.org/WAI/WCAG21/Understanding/label-in-name#dfn-label) that include [text](https://www.w3.org/WAI/WCAG21/Understanding/label-in-name#dfn-text) or [images of text](https://www.w3.org/WAI/WCAG21/Understanding/label-in-name#dfn-image-of-text), the [name](https://www.w3.org/WAI/WCAG21/Understanding/label-in-name#dfn-name) contains the text that is presented visually.  **Note**  A best practice is to have the text of the label at the start of the name. | Migration, UI | ePROM migration best practice is to ensure core wording is maintained, which would be applicable to response options and so the words which visibly ‘label’ the response type component would also be the word associated with the component programmatically.  This should also be the case for any user interface components in the system as a whole that have labels that include text or images of text. | Maintain existing best practices. |
| A | [2.5.4 Motion Actuation](https://www.w3.org/WAI/WCAG21/Understanding/motion-actuation)  [Functionality](https://www.w3.org/WAI/WCAG21/Understanding/motion-actuation#dfn-functionality) that can be operated by device motion or user motion can also be operated by [user interface components](https://www.w3.org/WAI/WCAG21/Understanding/motion-actuation#dfn-user-interface-component) and responding to the motion can be disabled to prevent accidental actuation, except when:  **Supported Interface**  The motion is used to operate functionality through an [accessibility supported](https://www.w3.org/WAI/WCAG21/Understanding/motion-actuation#dfn-accessibility-supported) interface;  **Essential**  The motion is [essential](https://www.w3.org/WAI/WCAG21/Understanding/motion-actuation#dfn-essential) for the function and doing so would invalidate the activity. | N/A | This does not apply to ePROMs as they do not use functionality triggered by moving a device (e.g., motion such as shaking). | N/A |
| AAA | [2.5.5 Target Size](https://www.w3.org/WAI/WCAG21/Understanding/target-size) (Enhanced)  The size of the [target](https://www.w3.org/WAI/WCAG21/Understanding/target-size#dfn-target) for [pointer inputs](https://www.w3.org/WAI/WCAG21/Understanding/target-size#dfn-pointer-input) is at least 44 by 44 [CSS pixels](https://www.w3.org/WAI/WCAG21/Understanding/target-size#dfn-css-pixel) except when:  **Equivalent**  The target is available through an equivalent link or control on the same page that is at least 44 by 44 CSS pixels;  **Inline**  The target is in a sentence or block of text;  **User Agent Control**  The size of the target is determined by the user agent and is not modified by the author;  **Essential**  A particular presentation of the target is [essential](https://www.w3.org/WAI/WCAG21/Understanding/target-size#dfn-essential) to the information being conveyed. | Migration, UI | See 2.5.8 | See 2.5.8 |
| AAA | [2.5.6 Concurrent Input Mechanisms](https://www.w3.org/WAI/WCAG21/Understanding/concurrent-input-mechanisms)  Web content does not restrict use of input modalities available on a platform except where the restriction is [essential](https://www.w3.org/WAI/WCAG21/Understanding/concurrent-input-mechanisms#dfn-essential), required to ensure the security of the content, or required to respect user settings.  Mobile specific  Users can enter information on mobile devices in multiple ways such as on-screen keyboard, Bluetooth keyboard, touch, and speech. | UI | This is not detailed in ePROM best practices. | eCOA systems should allow for all content to be operable though different input mechanisms. |
| AA | [2.5.7 Dragging Movements](https://www.w3.org/WAI/WCAG22/Understanding/dragging-movements)  All [functionality](https://www.w3.org/WAI/WCAG22/Understanding/dragging-movements#dfn-functionality) that uses a [dragging movement](https://www.w3.org/WAI/WCAG22/Understanding/dragging-movements#dfn-dragging-movement) for operation can be achieved by a [single pointer](https://www.w3.org/WAI/WCAG22/Understanding/dragging-movements#dfn-single-pointer) without dragging, unless dragging is [essential](https://www.w3.org/WAI/WCAG22/Understanding/dragging-movements#dfn-essential) or the functionality is determined by the [user agent](https://www.w3.org/WAI/WCAG22/Understanding/dragging-movements#dfn-user-agent) and not modified by the author.  **Note**  This requirement applies to web content that interprets pointer actions (i.e. this does not apply to actions that are required to operate the user agent or assistive technology). | Core System Functionality | Current ePROM best practice for interacting with, and operating the VAS is to allow the user to select a point on the line (their response) by either tapping or sliding (or both), and so meets this criterion as both options to operate the response scale are possible. | Maintain existing best practices. |
| AA | [2.5.8 Target Size (Minimum)](https://www.w3.org/WAI/WCAG22/Understanding/target-size-minimum)  The size of the [target](https://www.w3.org/WAI/WCAG22/Understanding/target-size-minimum#dfn-target) for [pointer inputs](https://www.w3.org/WAI/WCAG22/Understanding/target-size-minimum#dfn-pointer-input) is at least 24 by 24 [CSS pixels](https://www.w3.org/WAI/WCAG22/Understanding/target-size-minimum#dfn-css-pixel), except where:   - **Spacing:** Undersized targets (those less than 24 by 24 CSS pixels) are positioned so that if a 24 CSS pixel diameter circle is centered on the [bounding box](https://www.w3.org/WAI/WCAG22/Understanding/target-size-minimum#dfn-minimum-bounding-box) of each, the circles do not intersect another target or the circle for another undersized target; - **Equivalent:** The function can be achieved through a different control on the same page that meets this criterion; - **Inline:** The target is in a sentence or its size is otherwise constrained by the line-height of non-target text; - **User agent control:** The size of the target is determined by the [user agent](https://www.w3.org/WAI/WCAG22/Understanding/target-size-minimum#dfn-user-agent) and is not modified by the author; - **Essential:** A particular [presentation](https://www.w3.org/WAI/WCAG22/Understanding/target-size-minimum#dfn-presentation) of the target is [essential](https://www.w3.org/WAI/WCAG22/Understanding/target-size-minimum#dfn-essential) or is legally required for the information being conveyed.   **Note**  Targets that allow for values to be selected spatially based on position within the target are considered one target for the purpose of the success criterion. Examples include sliders, color pickers displaying a gradient of colors, or editable areas where you position the cursor.  **Note**  For inline targets the line-height should be interpreted as perpendicular to the flow of text. For example, in a language displayed vertically, the line-height would be horizontal.  Mobile specific  The high resolution of mobile devices means that many interactive elements can be shown together on a small screen. But these elements must be big enough and have enough distance from each other so that users can safely target them by touch.  Best practices for touch target size include the following:   - Ensuring that touch targets are at least 9 mm high by 9 mm wide. - Ensuring that touch targets close to the minimum size are surrounded by a small amount of inactive space.   *Note:* This size is not dependent on the screen size, device or resolution. Screen magnification should not need to be used to obtain this size, because magnifying the screen often introduces the need to pan horizontally as well as vertically, which can decrease usability. | Migration, UI | Current ePROM best practices are that the entire NRS must be visible on one screen without scrolling, that each number response option is the same size, and the NRS does not wrap across lines. Further, on mobile devices it is common to lock the orientation to portrait (as detailed in 1.3.4). Given that changing this would essentially change the information or functionality of the content, the criterion details that the target does not need to meet the minimum size detailed as the requirements are essential to the scale type. This is likely to be especially applicable to mobile devices where screen sizes are small. | eCOA systems should ensure that all eCOA systems should ensure that all interface components have a target size of 24 by 24 CSS pixels – unless a smaller size is essential to the information being conveyed. Specifically for mobile devices, it is recommended that touch targets are at least 9 mm high by 9 mm wide, and that those close to the minimum size are surrounded by a small amount of inactive space.  In instances where the above are not possible, consider the use of devices with larger screen sizes or enabling orientation changes (see 1.3.4.).  It is recommended that future research is conducted to evaluate different presentations of the NRS (e.g., a vertical presentation), that could optimize usability and accessibility, to ensure measurement comparability is maintained. |
| *3.1 Make text content readable and understandable.* | |  |  |  |
| A | [3.1.1 Language of Page](https://www.w3.org/WAI/WCAG21/Understanding/language-of-page)  The default [human language](https://www.w3.org/WAI/WCAG21/Understanding/language-of-page#dfn-human-language) of each [Web page](https://www.w3.org/WAI/WCAG21/Understanding/language-of-page#dfn-web-page) can be [programmatically determined](https://www.w3.org/WAI/WCAG21/Understanding/language-of-page#dfn-programmatically-determined). | UI | When ePROMs are used in global studies, they will be translated into the specific locales required and the correct locale will be assigned to each user on the device to ensure they receive the localisation of the ePROM appropriate to them. The platform should also be set-up so the language can be programmatically identified by assistive technologies. | Ensure the ePRO system enables the programmatic identification of a page’s language. |
| AA | [3.1.2 Language of Parts](https://www.w3.org/WAI/WCAG21/Understanding/language-of-parts) | N/A | This does not apply to ePROMs but is relevant to the actual development of a PROM which should be ‘fit-for-purpose' and would not contain changes in language beyond those listed as exceptions. | N/A |
| AAA | [3.1.3 Unusual Words](https://www.w3.org/WAI/WCAG21/Understanding/unusual-words)  A [mechanism](https://www.w3.org/WAI/WCAG21/Understanding/unusual-words#dfn-mechanism) is available for identifying specific definitions of words or phrases [used in an unusual or restricted way](https://www.w3.org/WAI/WCAG21/Understanding/unusual-words#dfn-used-in-an-unusual-or-restricted-way), including [idioms](https://www.w3.org/WAI/WCAG21/Understanding/unusual-words#dfn-idiom) and [jargon](https://www.w3.org/WAI/WCAG21/Understanding/unusual-words#dfn-jargon). | N/A | This does not apply to ePROMs but is relevant to the actual development of a PROM which should be ‘fit-for-purpose' in the target population. | N/A |
| AAA | [3.1.4 Abbreviations](https://www.w3.org/WAI/WCAG21/Understanding/abbreviations)  A [mechanism](https://www.w3.org/WAI/WCAG21/Understanding/abbreviations#dfn-mechanism) for identifying the expanded form or meaning of [abbreviations](https://www.w3.org/WAI/WCAG21/Understanding/abbreviations#dfn-abbreviation) is available. | N/A | This does not apply to ePRO but is relevant to the actual development of a PROM which should be ‘fit-for-purpose' in the target population. | N/A |
| AAA | [3.1.5 Reading Level](https://www.w3.org/WAI/WCAG21/Understanding/reading-level) When text requires reading ability more advanced than the [lower secondary education level](https://www.w3.org/WAI/WCAG21/Understanding/reading-level#dfn-lower-secondary-education-level) after removal of proper names and titles, [supplemental content](https://www.w3.org/WAI/WCAG21/Understanding/reading-level#dfn-supplemental-content), or a version that does not require reading ability more advanced than the lower secondary education level, is available. | N/A | This does not apply to ePRO but is relevant to the actual development of a PROM which should be ‘fit-for-purpose' in the target population. | N/A |
| AAA | [3.1.6 Pronunciation](https://www.w3.org/WAI/WCAG21/Understanding/pronunciation)  A [mechanism](https://www.w3.org/WAI/WCAG21/Understanding/pronunciation#dfn-mechanism) is available for identifying specific pronunciation of words where meaning of the words, in context, is ambiguous without knowing the pronunciation. | N/A | This does not apply to ePRO but is relevant to the actual development of a COA which should be ‘fit-for-purpose' in the target population. | N/A |
| *3.2 Make Web pages appear and operate in predictable ways.* | |  |  |  |
| A | [3.2.1 On Focus](https://www.w3.org/WAI/WCAG21/Understanding/on-focus) When any [user interface component](https://www.w3.org/WAI/WCAG21/Understanding/on-focus#dfn-user-interface-component) receives focus, it does not initiate a [change of context](https://www.w3.org/WAI/WCAG21/Understanding/on-focus#dfn-changes-of-context). | Core system functionality | Current ePROM best practice is that there should be no auto-advance to the next screen on selection of a response option, without the respondent selecting the applicable navigation button. | Maintain existing best practices. |
| A | [3.2.2 On Input](https://www.w3.org/WAI/WCAG21/Understanding/on-input)  Changing the setting of any [user interface component](https://www.w3.org/WAI/WCAG21/Understanding/on-input#dfn-user-interface-component) does not automatically cause a [change of context](https://www.w3.org/WAI/WCAG21/Understanding/on-input#dfn-changes-of-context) unless the user has been advised of the behavior before using the component. | Core system functionality | Current ePROM best practices is that there should be no auto-advance to the next screen on selection of a response option, without the respondent selecting the applicable navigation button, and that there should be a final save and submit screen that informs users when they have reached the end of a measure and can go back and review or change any responses before saving and submitting, at which point they will no longer be able to. | Maintain existing best practices. |
| AA | [3.2.3 Consistent Navigation](https://www.w3.org/WAI/WCAG21/Understanding/consistent-navigation)  Navigational mechanisms that are repeated on multiple [Web pages](https://www.w3.org/WAI/WCAG21/Understanding/consistent-navigation#dfn-web-page) within a [set of Web pages](https://www.w3.org/WAI/WCAG21/Understanding/consistent-navigation#dfn-set-of-web-pages) occur in the [same relative order](https://www.w3.org/WAI/WCAG21/Understanding/consistent-navigation#dfn-same-relative-order) each time they are repeated, unless a change is initiated by the user. | UI | Current ePROM best practice is that navigation buttons should be placed consistently on each screen. | Maintain existing best practices. |
| AA | [3.2.4 Consistent Identification](https://www.w3.org/WAI/WCAG21/Understanding/consistent-identification)  Components that have the [same functionality](https://www.w3.org/WAI/WCAG21/Understanding/consistent-identification#dfn-same-functionality) within a [set of Web pages](https://www.w3.org/WAI/WCAG21/Understanding/consistent-identification#dfn-set-of-web-pages) are identified consistently.  Mobile specific  Elements that trigger changes should be sufficiently distinct to be clearly distinguishable from non-actionable elements (content, status information, etc). Providing a clear indication that elements are actionable is relevant for web and native mobile applications that have actionable elements like buttons or links, especially in interaction modes where actionable elements are commonly detected visually (touch and mouse use). Interactive elements must also be detectable by users who rely on a programmatically determined accessible name (e.g. screen reader users).  Visual users who interact with content using touch or visual cursors (e.g. mice, touchpads, joysticks) should be able to clearly distinguish actionable elements such as links or buttons. Existing interface design conventions are aimed at indicating that these visual elements are actionable. The principle of redundant coding ensures that elements are indicated as actionable by more than one distinguishing visual feature. Following these conventions benefits all users, but especially users with vision impairments.  Visual features that can set an actionable element apart include shape, color, style, positioning, text label for an action, and conventional iconography.  Examples of distinguishing features:   1. Conventional shape: Button shape (rounded corners, drop shadows), checkbox, select rectangle with arrow pointing downwards 2. Iconography: conventional visual icons (question mark, home icon, burger icon for menu, floppy disk for save, back arrow, etc) 3. Color offset: shape with different background color to distinguish the element from the page background, different text color 4. Conventional style: Underlined text for links, color for links   Conventional positioning: Commonly used position such as a top left position for back button (iOS), position of menu items within left-aligned lists in drop-down menus for navigation. | UI | This is not detailed in ePROM best practices, but assumed to be part of the UI.  This can be challenging when sometimes copyright holders dictate how they would like the buttons to be labelled; for example, some want a “Next” button on the first screen and others want a “Start” button which could lead to different navigation button names across measures within a study.  If vendors are using operating system native components, this could also lead to inconsistent identification, as IOS and Android sometimes use different labels for navigation buttons. With the increase in BYOD and the potential that a user could swap to a different operating system within a study (e.g., if they get a new device), this could be problematic and cause confusion, in addition to if web-back ups are used that also have different identification. | Use consistent identification and labeling of functional components that appear repeatedly throughout the eCOA system. If icons or other non-text items also have the same functionality, these should be consistent as well as their text alternatives.  It can be argued that the functional components are part of the eCOA vendors product, and not the actual measure, and so copyright holders should not dictate this identification, especially given the confusion this might cause for users if multiple measures use different identifications. |
| AAA | [3.2.5 Change on Request](https://www.w3.org/WAI/WCAG21/Understanding/change-on-request)  [Changes of context](https://www.w3.org/WAI/WCAG21/Understanding/change-on-request#dfn-changes-of-context) are initiated only by user request or a [mechanism](https://www.w3.org/WAI/WCAG21/Understanding/change-on-request#dfn-mechanism) is available to turn off such changes. | Core system functionality | Current ePROM best practices are that there should be no auto-advance to the next screen on selection of a response option, without the respondent selecting the applicable navigation button, and that there should be a final save and submit screen that informs users when they have reached the end of the measure that they can go back and review or change any responses before saving and submitting, at which point they will no longer be able to and the user will control if they go back or save and submit though their navigation selection. | Maintain existing best practices. |
| A | [3.2.6 Consistent Help](https://www.w3.org/WAI/WCAG22/Understanding/consistent-help)  If a [Web page](https://www.w3.org/WAI/WCAG22/Understanding/consistent-help#dfn-web-page) contains any of the following help [mechanisms](https://www.w3.org/WAI/WCAG22/Understanding/consistent-help#dfn-mechanism), and those mechanisms are repeated on multiple Web pages within a [set of Web pages](https://www.w3.org/WAI/WCAG22/Understanding/consistent-help#dfn-set-of-web-pages), they occur in the same order relative to other page content, unless a change is initiated by the user:   - Human contact details; - Human contact mechanism; - Self-help option; - A fully automated contact mechanism.   **Note**  Help mechanisms may be provided directly on the page, or may be provided via a direct link to a different page containing the information.  **Note**  For this Success Criterion, "the same order relative to other page content" can be thought of as how the content is ordered when the page is serialized. The visual position of a help mechanism is likely to be consistent across pages for the same page variation (e.g., CSS break-point). The user can initiate a change, such as changing the page's zoom or orientation, which may trigger a different page variation. This criterion is concerned with relative order across pages displayed in the same page variation (e.g., same zoom level and orientation). | Core System Functionality | ePROMs do not include the content types covered by this success criteria; however, ePRO systems should provide contact details for users to access help and so should be mindful of this. | N/A |
| *3.3 Help users avoid and correct mistakes.* | |  |  |  |
| A | [3.3.1 Error Identification](https://www.w3.org/WAI/WCAG21/Understanding/error-identification) If an [input error](https://www.w3.org/WAI/WCAG21/Understanding/error-identification#dfn-input-error) is automatically detected, the item that is in error is identified and the error is described to the user in text. | Core system functionality | Current ePROM best practice is to prevent out-of-range responses which can be programmed by limiting out of range data and automatically detecting when this occurs by the user, and informing the user of an error. Detail regarding the recommended behavior to communicate the out-of-range or illogical response is not provided (e.g., how users are informed that their response was out-of-range and therefore not accepted by the system), and would depend on the content type (e.g., are they simply told it is out of range or should detail of the range be provided?). | Maintain existing best practices, and expand with recommendation on how detail of the error should be communicated to users (i.e., provide the available programmed range, or just inform the user of an error and how to action it). |
| A | [3.3.2 Labels or Instructions](https://www.w3.org/WAI/WCAG21/Understanding/labels-or-instructions)  [Labels](https://www.w3.org/WAI/WCAG21/Understanding/labels-or-instructions#dfn-label) or instructions are provided when content requires user input.  Mobile specific  The ability to provide control via custom touchscreen and device manipulation gestures can help developers create efficient new interfaces. However, for many people, custom gestures can be a challenge to discover, perform and remember.  Therefore, instructions (e.g. overlays, tooltips, tutorials, etc.) should be provided to explain what gestures can be used to control a given interface and whether there are alternatives. To be effective, the instructions should, themselves, be easily discoverable and accessible. The instructions should also be available anytime the user needs them, not just on first use, though on first use they may be made more apparent through highlighting or some other mechanism.  On some mobile devices, the standard keyboard can be customized in the device settings and additional custom keyboards can be installed. Some mobile devices also provide different virtual keyboards depending on the type of data entry. This can be set by the user or can be set to a specific keyboard. For example, using the different HTML5 form field controls (see [Method Editor API](https://www.w3.org/TR/ime-api/)) on a website will show different keyboards automatically when users are entering in information into that field. Setting the type of keyboard helps prevent errors and ensures formats are correct but can be confusing for people who are using a screen reader when there are subtle changes in the keyboard. | Migration, UI | Current ePROM best practice to ensure a faithful migration means that there should not be any changes to the measure wording (apart from those to align with the new mode of administration) and that all information required to fully understand an item and select the desired response must be included. This would include each response option in the electronic format having an appropriate label so they know what they are selecting – i.e., any option for user input provided to a user should have a label.  Providing on-device instruction and training on system use is also a best practice and should include sample questions composed of the input types they will encounter during the study. | Maintain existing best practices, and expand to include recommendation that when a virtual keyboard is used, the keyboard presented for a given item is relevant to the response format (e.g., present a number pad for numeric entry). |
| AA | [3.3.3 Error Suggestion](https://www.w3.org/WAI/WCAG21/Understanding/error-suggestion)  If an [input error](https://www.w3.org/WAI/WCAG21/Understanding/error-suggestion#dfn-input-error) is automatically detected and suggestions for correction are known, then the suggestions are provided to the user, unless it would jeopardize the security or purpose of the content. | Core System Functionality | As detailed in 3.3.1, current ePROM best practice is to prevent out-of-range responses which can be programmed by limiting out of range data and automatically detecting when this occurs from the user; in this case, the user may be informed of the range but not a more specific suggestion as this could jeopardize the purpose of the content (i.e. collecting accurate self-reports from the participant). | N/A |
| AA | [3.3.4 Error Prevention (Legal, Financial, Data)](https://www.w3.org/WAI/WCAG21/Understanding/error-prevention-legal-financial-data)  For [Web pages](https://www.w3.org/WAI/WCAG21/Understanding/error-prevention-legal-financial-data#dfn-web-page) that cause [legal commitments](https://www.w3.org/WAI/WCAG21/Understanding/error-prevention-legal-financial-data#dfn-legal-commitments) or financial transactions for the user to occur, that modify or delete [user-controllable](https://www.w3.org/WAI/WCAG21/Understanding/error-prevention-legal-financial-data#dfn-user-controllable) data in data storage systems, or that submit user test responses, at least one of the following is true:  **Reversible**  Submissions are reversible.  **Checked**  Data entered by the user is checked for input errors and the user is provided an opportunity to correct them.  **Confirmed**  A mechanism is available for reviewing, confirming, and correcting information before finalizing the submission. | N/A | This does not apply to ePROMs as they do not include this type of data. | N/A |
| AAA | [3.3.5 Help](https://www.w3.org/WAI/WCAG21/Understanding/help)  [Context-sensitive help](https://www.w3.org/WAI/WCAG21/Understanding/help#dfn-context-sensitive-help) is available. | Core system functionality, UI | Providing training on system use is an ePROM best practice, and assistance with understanding of the content of the PROM itself is relevant to the actual development of the measure by the author, which should be ‘fit-for-purpose' in the target population.^5^  This criterion details that context-sensitive help only needs to be provided when a label is not sufficient to describe the functionality, and so any UI components (e.g., “Next” button) should have labels that achieve this | Maintain existing best practices. |
| AAA | [3.3.6 Error Prevention (All)](https://www.w3.org/WAI/WCAG21/Understanding/error-prevention-all)  For [Web pages](https://www.w3.org/WAI/WCAG21/Understanding/error-prevention-all#dfn-web-page) that require the user to submit information, at least one of the following is true:  **Reversible**  Submissions are reversible.  **Checked**  Data entered by the user is checked for input errors and the user is provided an opportunity to correct them.  **Confirmed**  A mechanism is available for reviewing, confirming, and correcting information before finalizing the submission. | Migration, Core system functionality | Current ePROM best practice is to include a final save and submit screen that informs respondents when they have reached the end of an ePROM and that they can go back and review and change any responses before submitting them, providing the opportunity to prevent errors. After the data is submitted, an error may still be corrected by contacting site personnel.  ePROM best practices also include capturing a data point for all items - this means if a respondent is allowed to skip that an edit-check is triggered to confirm their intention to skip the question without providing a response. ePROM best practices also include the use of edit-checks for out-of-range or illogical responses by programming a pop-up message when such an entry occurs. Both best practices check for input errors and provide the opportunity for the user to correct them. | Maintain existing best practices. |
| A | [3.3.7 Redundant Entry](https://www.w3.org/WAI/WCAG22/Understanding/redundant-entry) | N/A | ePROMs do not require the respondent to enter the same information multiple times in the same process. | N/A |
| AA | [3.3.8 Accessible Authentication (Minimum)](https://www.w3.org/WAI/WCAG22/Understanding/accessible-authentication-minimum)  A [cognitive function test](https://www.w3.org/WAI/WCAG22/Understanding/accessible-authentication-minimum#dfn-cognitive-function-test) (such as remembering a password or solving a puzzle) is not required for any step in an authentication [process](https://www.w3.org/WAI/WCAG22/Understanding/accessible-authentication-minimum#dfn-process) unless that step provides at least one of the following:  **Alternative**  Another authentication method that does not rely on a cognitive function test.  **Mechanism**  A [mechanism](https://www.w3.org/WAI/WCAG22/Understanding/accessible-authentication-minimum#dfn-mechanism) is available to assist the user in completing the cognitive function test.  **Object Recognition**  The cognitive function test is to recognize objects.  **Personal Content**  The cognitive function test is to identify [non-text content](https://www.w3.org/WAI/WCAG22/Understanding/accessible-authentication-minimum#dfn-non-text-content) the user provided to the Web site.  **Note**  "Object recognition" and "Personal content" may be represented by images, video, or audio.  **Note**  Examples of mechanisms that satisfy this criterion include:   1. support for password entry by password managers to reduce memory need, and 2. copy and paste to reduce the cognitive burden of re-typing. | Core system functionality | This criterion is not relevant to the ePROM itself; however, ePRO systems should be secure and require a password to login, an input type that is covered, and so should be mindful of this. | N/A |
| AAA | [3.3.9 Accessible Authentication (Enhanced)](https://www.w3.org/WAI/WCAG22/Understanding/accessible-authentication-enhanced) | Core system functionality | See 3.3.8 | N/A |
| *4.1 Maximize compatibility with current and future user agents, including assistive technologies.* | |  |  |  |
| A | [4.1.2 Name, Role, Value](https://www.w3.org/WAI/WCAG21/Understanding/name-role-value)  For all [user interface components](https://www.w3.org/WAI/WCAG21/Understanding/name-role-value#dfn-user-interface-component) (including but not limited to: form elements, links and components generated by scripts), the [name](https://www.w3.org/WAI/WCAG21/Understanding/name-role-value#dfn-name) and [role](https://www.w3.org/WAI/WCAG21/Understanding/name-role-value#dfn-role) can be [programmatically determined](https://www.w3.org/WAI/WCAG21/Understanding/name-role-value#dfn-programmatically-determined); states, properties, and values that can be set by the user can be [programmatically set](https://www.w3.org/WAI/WCAG21/Understanding/name-role-value#dfn-programmatically-set); and notification of changes to these items is available to [user agents](https://www.w3.org/WAI/WCAG21/Understanding/name-role-value#dfn-user-agent), including [assistive technologies](https://www.w3.org/WAI/WCAG21/Understanding/name-role-value#dfn-assistive-technology).  **Note**  This success criterion is primarily for Web authors who develop or script their own user interface components. For example, standard HTML controls already meet this success criterion when used according to specification. | UI, Core system functionality | This is not directly referred to in existing ePROM best practices but the core system functionality should support this.  However, detail is provided, that when navigating between screens that have already been encountered and a response option selected, then this previous response selection should be clear to the user, including identification by assistive technologies. Again, this would be challenging for the VAS given the selection is a visual point, and the response not associated with an identifiable label to the user. | The ePRO system should be able to support the programmatic determination of the name and role of user interface components, and any changes to the state of these elements should be identifiable by all.  For new PROMs, do not include visual analogue scales as a response type. |
| AA | [4.1.3 Status Messages](https://www.w3.org/WAI/WCAG21/Understanding/status-messages)  In content implemented using markup languages, [status messages](https://www.w3.org/WAI/WCAG21/Understanding/status-messages#dfn-status-message) can be [programmatically determined](https://www.w3.org/WAI/WCAG21/Understanding/status-messages#dfn-programmatically-determined) through [role](https://www.w3.org/WAI/WCAG21/Understanding/status-messages#dfn-role) or properties such that they can be presented to the user by [assistive technologies](https://www.w3.org/WAI/WCAG21/Understanding/status-messages#dfn-assistive-technology) without receiving focus. | UI, Core system functionality | Current ePROM best practices are to capture a data point for all items. If a respondent is not allowed to skip an item, a pop-up message should be programmed to appear if they press the “Next” button without selecting a response, indicating the requirement to provide a response before being able to proceed. If respondents are allowed to skip an item, then an edit-check should be triggered to confirm their intention so skip the question without providing a response. In both these scenarios, these would not be classified as a status message as the message is displayed in a dialog that takes focus and this change in context would be announced by the screen reader.  Another ePROM best practice is to prevent out-of-range or illogical responses by programming a pop-up message. As above this would not classify as a status message.  If these functionalities were to be implemented, not as a pop-up displayed in a dialog and a change in context, but by adding this new information on the actual page itself which is not necessarily given focus, then this success criteria would be relevant. | Maintain existing best practices.  If messages displayed to the user are not going to be implemented in a dialog but instead by adding new information to the page itself, then the ePRO system should ensure that they can be programmatically determined and presented to users of assistive technologies without receiving focus. |

* Migration = the success criteria are directly related to best practices for the migration from paper and electronic implementation of a PROM; Core system functionality = the success criteria are related to functionality the system should support to enable the electronic implementation of PROMS; UI = the success criteria are related to the ePRO provider’s platform as a whole, rather than solely to the ePROM and should be created as a platform standard; N/A = Not Applicable to ePROMs

Of note, in WCAG 2.2, success criterion 4.1.1 was deemed obsolete and removed but numbering was not updated so as not to cause confusion and compatibility with previous criterion.

Current ePROM best practices refers to Mowlem et al., 2024 ^6^
